# The Safety of Telerehabilitation: Systematic Review

**DOI:** 10.2196/68681

**Published:** 2025-07-09

**Authors:** Hila Shnitzer, Josh Chan, Thomas Yau, McKyla McIntyre, Angie Andreoli, Ailene Kua, Mark Bayley, Carl Froilan Leochico, Meiqi Guo, Sarah Munce

**Affiliations:** 1 Michael G. DeGroote School of Medicine, McMaster University, Hamilton, ON, Canada; 2 Western University, London, ON, Canada; 3 Temerty Faculty of Medicine, University of Toronto, Toronto, ON, Canada; 4 Toronto Rehabilitation Institute, University Health Network, 550 University Avenue, Toronto, ON, M5G 2A2, Canada, 1 4165973422; 5 KITE Research Institute, Toronto Rehabilitation Institute, University Health Network, Toronto, ON, Canada; 6 St. Luke’s Medical Center, Global City, Philippines; 7 Philippine General Hospital, University of the Philippines Manila, Manila, Philippines; 8 Rehabilitation Sciences Institute, University of Toronto, Toronto, ON, Canada; 9 Bloorview Research Institute, Holland Bloorview Kids Rehabilitation Hospital, Toronto, ON, Canada

**Keywords:** telerehabilitation, telehealth, remote health care, safety, exercise, telemedicine, adverse events, rehabilitation, digital health, patient safety, exercise therapy, videoconferencing

## Abstract

**Background:**

Telerehabilitation involves the delivery of rehabilitation services over a distance through communication technologies. In contrast to traditional in-person rehabilitation, telerehabilitation can help overcome barriers including geographic distance and facility use. There is evidence to suggest that telerehabilitation can lead to increased patient engagement and adherence to treatment plans. However, limited research exists on the association of telerehabilitation with adverse events, potentially hindering its broader adoption and use in health care.

**Objectives:**

This systematic review of randomized controlled trials aims to summarize existing research on adverse events related to telerehabilitation delivery.

**Methods:**

This review was conducted according to the methodological framework outlined by the Joanna Briggs Institute. Studies were identified from MEDLINE ALL, Embase, APA PsycINFO, CENTRAL, and CINAHL. Included studies were randomized controlled trials published between 2013 and 2023, written in English, and had no geographic or delivery mode restrictions. Data extraction used the TIDieR (Template for Intervention Description and Replication) framework, along with authors, publication year, sample size, specific telerehabilitation modes, and the incidence, type, severity, and relatedness of reported adverse events. Methodological quality was assessed using the Cochrane risk of bias tool, and the certainty of evidence was evaluated using the Grading of Recommendations Assessment, Development, and Evaluation tool.

**Results:**

Search results identified 9022 references, of which 37 randomized controlled trials met the criteria for inclusion. There were a total of 3166 participants, with a mean age of 57.4 (SD 11.3) years, and 1023 (32.3%) being female participants. Various delivery modes were used, with videoconferencing emerging as the most frequently used method. A total of 201 adverse events were recorded during 65,352 sessions (0.31% or 3.1 per 1000 sessions). These events were predominantly physical (eg, falls and palpitations), nonserious or mild, and not directly attributed to the telerehabilitation intervention. Additionally, 34 (92%) of included studies implemented various safety practices including vital sign monitoring, safety checklists, and scheduled check-ins with study personnel.

**Conclusions:**

This review demonstrates that telerehabilitation exhibits a generally safe profile as an alternative to in-person rehabilitation, with most reported adverse events being rare, nonserious or mild, and unrelated to telerehabilitation protocols. However, more extensive research with detailed reporting on adverse event characteristics is needed. Moreover, future research should evaluate the effectiveness of different safety practices and their association with adverse events. An enhanced understanding of potential risks in telerehabilitation can foster broader adoption while ensuring its safe implementation among health care providers and patients.

## Introduction

Telerehabilitation refers to the delivery of rehabilitation services through communication technologies, allowing patients to connect with therapists remotely without needing to be in the same physical space [1,2]. These services can be provided synchronously, with live therapist interactions, or asynchronously, where patients perform therapeutic activities independently [3,4]. Telerehabilitation has been successfully implemented across various rehabilitation fields, including physical therapy, occupational therapy, and speech language therapy, and its benefits have been described in neurological populations as well as during the early stages of the COVID-19 pandemic [5,6].

In recent years, telerehabilitation has gained rapid popularity due to its ability to overcome barriers such as geographic distance, mobility limitations, and scheduling constraints while also reducing costs by minimizing facility and resource use [7,8]. For example, the American Physical Therapy Association found that telerehabilitation is superior to in-person rehabilitation with respect to adherence for certain health conditions [9]. The surge in utility was accelerated by the COVID-19 pandemic, which increased the demand for remote and easily accessible health care solutions [10].

Despite these advantages, telerehabilitation is not without risks. For instance, patients could incur a burn while cooking during a video-based occupational therapy session or sustain soft-tissue injuries from overexercising during an asynchronous text or phone-based physiotherapy session. A mixed methods study on individuals with chronic knee pain found that 36% of participants initially expressed negative feelings about telerehabilitation due to doubts about its effectiveness, the inability of physiotherapists to physically assess them, and concerns about difficulty communicating using technology [11].

Assessing the safety of telerehabilitation is important for guiding decisions made by patients, providers, and funders. For patients, a clearer understanding of its safety can offer reassurance and encourage broader adoption of telerehabilitation [12]. Providers rely on safety data to tailor treatments to individual conditions and refine protocols to minimize adverse events [13]. Funders rely on this information to justify coverage decisions and guide the allocation of resources and financial support [14]. Additionally, patients in rehabilitation often have physical or cognitive deficits (eg, a patient with hemiplegia and memory deficits from brain injury) that make them more vulnerable to risks compared to general telemedicine users, making the evaluation of telerehabilitation safety even more critical for preventing exacerbating existing conditions or for introducing new risks [15].

A scoping review by Yau et al [16] explored the association between telerehabilitation and adverse events. They found that adverse events were reported in 0.3% of sessions, with most being physical, nonserious or mild, and unrelated to the telerehabilitation intervention. While scoping reviews identify broad patterns in the literature, they do not offer in-depth assessments of study outcomes or assess the quality of individual studies. A systematic review can follow a scoping review when a more detailed and specific understanding of outcomes is required. Therefore, the objective of this systematic review is to assess the safety of telerehabilitation by examining the incidence, type, severity, and relatedness of adverse events reported in randomized controlled trials. Moving from a scoping review to a systematic review aligns with the purpose of a scoping review, as it builds on the initial broad exploration by transitioning into a more detailed examination of specific research questions [17].

## Methods

This systematic review was registered with PROSPERO (CRD42024518902) and drafted in accordance with the PRISMA (Preferred Reporting Items for Systematic Reviews and Meta-Analyses) statement [18]. A completed PRISMA checklist is shown in Checklist 1.

### Eligibility Criteria

The inclusion criteria for this review encompassed randomized controlled trials of any type (eg, parallel, crossover, and cluster) that (1) evaluated telerehabilitation interventions, (2) reported on any adverse events that occurred, and (3) were published between 2013 and 2023, as 2013 marked the beginning of significant advancements and widespread adoption of video communication technologies [19]. Studies that assessed a variety of patient populations (eg, cardiac rehabilitation and postoperative rehabilitation) and modes (eg, asynchronous, synchronous, and hybrid) of telerehabilitation were included. We excluded studies that (1) only provided home-based exercise programs without interactions from a therapist to adjust or monitor the exercise program and (2) were not written in English.

### Search Strategy and Information Sources

Literature search strategies were developed by an experienced health sciences librarian with input from the investigators. Search terms included MeSH, EMTREE terms, American Psychological Association thesaurus terms, and CINAHL headings, and text words related to telerehabilitation and adverse events. The search strategy was tested in MEDLINE ALL and reviewed by the research team. Once the MEDLINE ALL test strategy was agreed upon, it was translated using the command language, controlled vocabulary, and appropriate search fields for each database and search platform. Studies were then identified by searching the following databases on the Ovid platform: MEDLINE ALL, Embase, APA PsycINFO, and CENTRAL. The CINAHL database was searched on the EBSCOhost platform. This search strategy was first implemented in the scoping review by Yau et al [16] from January 2013 to June 2023 on June 26, 2023. The current research team then identified the randomized controlled trials from the extracted studies and confirmed their eligibility for this systematic review. On January 8, 2024, the search was repeated and extended to include studies published between June 2023 and December 2023. The same search strategy was used, although it was restricted to randomized controlled trials. The search strategy used for each database can be viewed in Multimedia Appendix 1.

### Study Selection and Data Extraction

Two reviewers (HS and JC) performed a pilot test and then completed the title and abstract screening independently and in duplicate. The full texts of potentially relevant papers were subsequently retrieved and screened (also independently and in duplicate). At all stages of the review, conflicts were resolved by using discussion to reach consensus or by a third party (TY).

Reviewers HS and JC then independently piloted the extraction form with a random sample of studies and made necessary revisions. Data extracted from publications included study characteristics, participant characteristics, intervention characteristics, and adverse events. Data extraction on intervention characteristics aligned with the TIDieR (Template for Intervention Description and Replication) framework [20]. Information on the incidence, type, severity, and relatedness of adverse events, safety measures implemented for the telerehabilitation interventions, as well as information on adverse events within control groups were also collected.

### Data Synthesis

Data from the extraction form were summarized descriptively using counts, proportions, and incidence rates where applicable. Subgroup analyses based on study characteristics (eg, rehabilitation population) were conducted to explore patterns in adverse event reporting. This involved aggregating reported event rates and comparing distributions across categories to identify potential trends in the findings. A meta-analysis was not conducted due to substantial heterogeneity in study populations, interventions, and outcome reporting.

### Risk of Bias Assessment

Five reviewers (HS, JC, TY, AK, and MG) independently evaluated the risk of bias in the included studies. Version 2 of the Cochrane risk-of-bias tool for randomized trials was used to appraise all randomized controlled trials [21]. For each included study, reviewers worked through individual domains within the tool and then assigned an overall risk-of-bias judgment. All studies were evaluated based on 5 key methodological elements: randomization process, blinding of participants and assessors, completeness of outcome data, validity of outcome measurement, and selection of reported results.

### Certainty of Evidence Evaluation

The certainty of the evidence was evaluated by JC using the Grading of Recommendations Assessment, Development, and Evaluation tool, which considers factors including study design, risk of bias, inconsistency in results, indirectness of evidence, imprecision of results, publication bias, effect size, plausible confounding, and dose-response gradient.

## Results

### Study Selection

**Figure 1.**
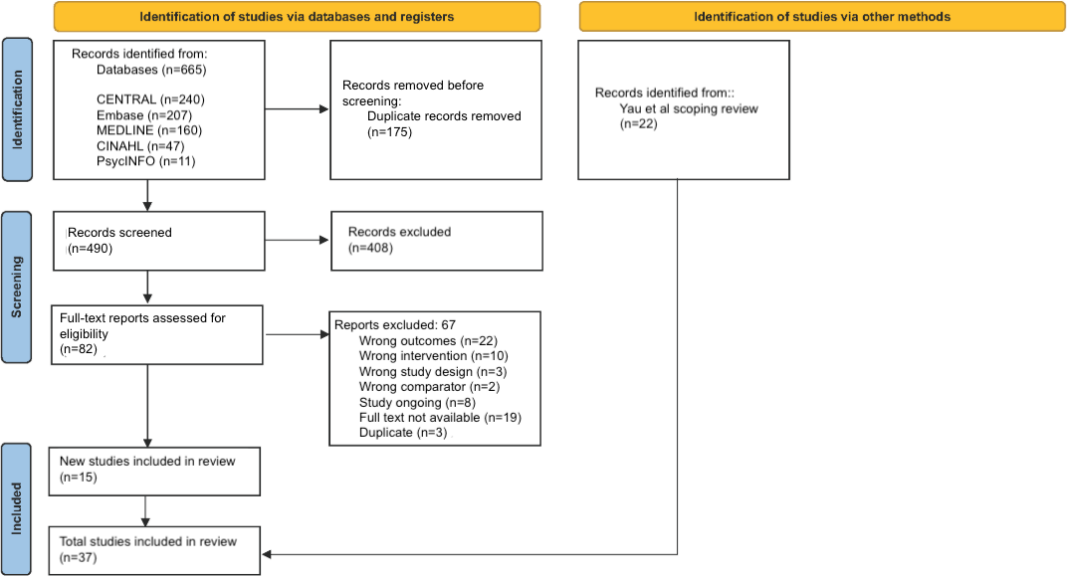
PRISMA (Preferred Reporting Items for Systematic Reviews and Meta-Analyses) flow diagram.

The search of all databases resulted in 9022 papers, of which 1008 were removed for duplication. A total of 7754 were excluded following a review of the title and abstract, and 223 were excluded following full-text review. The selection process with reasons for paper exclusion at each stage is presented in Figure 1. Ultimately, 37 papers met the inclusion criteria for data extraction and risk of bias assessment (Figure 1).

### Description of the Included Studies

All of the included papers were randomized controlled trials. The studies were conducted in 22 countries, with the 3 most common countries being China [22-27], the United States [28-32], and Australia [33-36]. The majority of papers were published following the COVID-19 pandemic, with 30 (81%) published from 2020 onward [22,23,25-32,34-53].

The included studies spanned a number of rehabilitation populations: 11 (30%) focused on musculoskeletal conditions involving interventions like balance, stretching, strengthening, and aerobic exercises [25,29,30,32,37,40,44,50,51,54,55]; 9 (24%) focused on cardiac conditions [23,24,28,33,39,43,46,53,56]; 6 (16%) focused on stroke [26,27,35,38,45,52]; 6 (16%) focused on other areas of neurorehabilitation [41,42,47,49,57,58]; 3 (8%) focused on pulmonary conditions [22,31,34]; 1 (3%) focused on cancer [48]; and 1 (3%) focused on burns [36] (Table 1).

**Table 1.** Distribution of rehabilitation populations.

Rehabilitation population	Studies, n (%)
Cardiac	9 (24)
Musculoskeletal	11 (30)
Neurological	6 (16)
Pulmonary	3 (8)
Stroke	6 (16)
Other	2 (6)

A total of 3166 participants were represented across the studies, with 1620 and 1546 in the intervention and control groups, respectively. There were 1023 female participants, accounting for 32% of the overall sample size. The participants in the intervention and control groups were similar at baseline in all studies. The mean age of participants was 57.4 (SD 11.3) years, ranging from 28.2 to 74.9 years. Multimedia Appendix 2 [22-32,34-59] provides a summary of the participant and intervention characteristics.

### Intervention Characteristics

In 68% (n=25) of the studies, the rehabilitation intervention was provided exclusively by a physiotherapist, occupational therapist, or their respective assistants. In total, 3 (8%) studies [23,27,48] used a physician to deliver the rehabilitation, which included exercise training and counseling, while 4 (11%) studies involved an interdisciplinary team. In 1 study examining pulmonary rehabilitation in participants with esophageal cancer [22], the interdisciplinary team included the head of the nursing department in thoracic surgery, at least 1 doctor who participated in the surgery, and 3 experienced nurses. Another study investigating cardiac rehabilitation in patients with heart failure in China [24] featured telerehabilitation delivered by a multidisciplinary team comprised of physiotherapists, cardiac nurses, and psychiatric nurses.

In 36 (97%) studies, participants completed therapy sessions from their respective homes, with only 1 study having participants complete rehabilitation at a fitness center [55]. In total, 32 (86%) rehabilitation protocols were entirely telerehabilitation-based, and 5 (13%) [28,34,45,56,57] included an in-person component. In 15 (40%) studies [23,24,26,28,29,31,33,34,36,38,39,41,44,47,51], therapists connected with patients synchronously, while 22 (60%) studies used asynchronous telerehabilitation. The intervention period ranged from 3 weeks to 1 year, with a 3-month protocol being the most common. Of the 27 (73%) studies that reported adherence, 2 achieved a 100% adherence rate [44,47].

In 29 (78%) studies, study personnel tailored the intervention to participants based on factors such as interest in physical exercise, rehabilitation goals, heart rate zones, and functional ability [23-29,31-34,36-39,42,45,47-58]. In total, 2 (5%) interventions carried out standardized testing before the intervention and used these results to personalize the rehabilitation protocol [50,53]. For example, Cerdan de Las Heras et al [50] used a baseline 6-minute walk test and an interview focused on participants’ daily activity to individualize the telerehabilitation program for participants with sarcoidosis. In total, 22 (59%) interventions used devices such as smartwatches, heart rate belts, electrocardiograms, blood pressure cuffs, pulse oximeters, pedometers, and various software tools to digitally monitor participants’ progress [22-24,27-31,33-35,37,38,40,41,45,46,49-51,54,56]. In 8 of these interventions, therapists used the collected data to adjust the rehabilitation protocol throughout the study period or to ensure that the protocol was being carried out safely [24,27,30,33,40,41,50,51].

Aside from vital sign monitoring, 54% (n=20) of the studies used additional risk mitigation strategies. In total, 11 (30%) studies implemented strategies to mitigate risks including scheduled follow-up times to review adverse events or general questions [22,23,29,30,35,43,46,48,53,55,58], on-call availability of study personnel for addressing concerns [31,39,44,47,52], educational sessions on pain and injury prevention [24,29,32,36], and the presence of caregivers to assist in case of emergencies [24,41]. A study by Capin et al [29], which provided high-intensity strength training and aerobic exercise to 13 survivors of COVID-19, described how the therapists optimized participants’ home environments to enhance safety, such as by instructing them to perform balance exercises near a bed to prevent falls.

### Control Characteristics

In 30 (81%) studies, the control group received usual care or center-based rehabilitation [22-24,26-28,30-39,42-47,49-56]. In total, 5 (13%) studies [40,41,48,51,58] provided control participants with booklets offering guidance on independent exercises. A total of 3 (8%) studies included education sessions or scheduled follow-ups with study personnel [25,29,44].

### Incidence of Adverse Events

**Figure 2.**
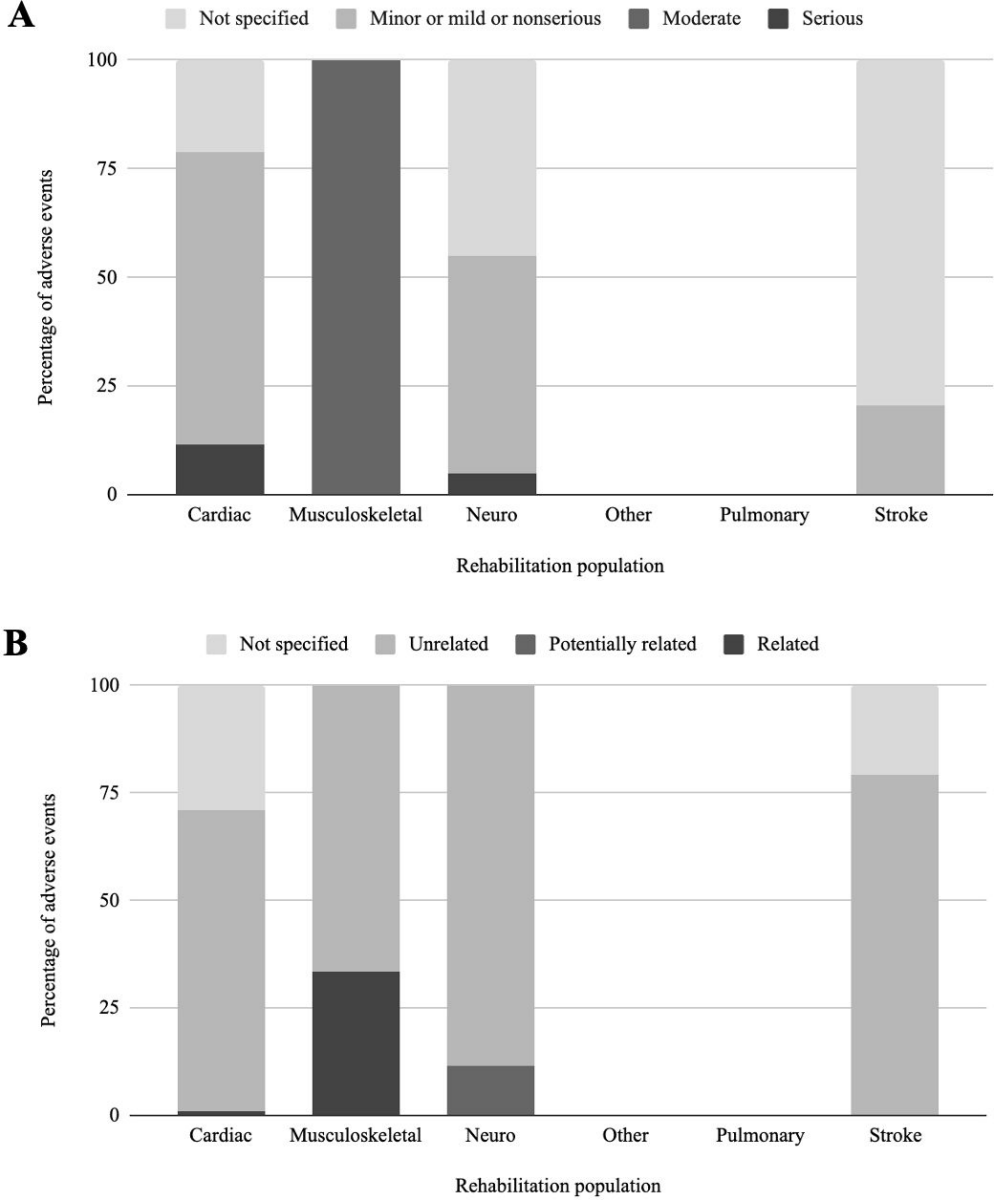
Adverse events. (A) Distribution of adverse event severity across rehabilitation populations. (B) Distribution of adverse event relatedness to the intervention across rehabilitation populations.

Multimedia Appendix 3 [22-32,34,35,37-49,51,53-59] summarizes the adverse events reported in the included telerehabilitation interventions. A total of 201 adverse events were recorded during 65,352 sessions (0.31 adverse events per 100 sessions). All studies reported information on the incidence of adverse events. In contrast, information on the type, severity, and relatedness of adverse events to the telerehabilitation intervention was infrequently documented. Among the 9 (24%) studies that provided severity details, 7 classified their adverse events as mild or nonserious, while the other 2 classified them as severe or serious. Mild adverse events included dizziness [26], fatigue [26,52], and dry eyes [26], with the most frequently cited being physical issues such as pain [41,51,52,57] and falls [28,41,52,58]. A study by Snoek et al [53], which delivered cardiac telerehabilitation asynchronously to 65 older patients, reported 3 serious adverse events over the intervention period, including vestibular disorder, vasovagal collapse, and knee injury during gardening that required surgery. These adverse events were ultimately determined to be unrelated to the telerehabilitation intervention. Of the 11 (30%) studies that documented relatedness, 8 [27,31,39,41,42,52,56,57] determined that their adverse events were unrelated to the intervention, while 3 studies for cardiac [28], neurological [57], and musculoskeletal [51] rehabilitation populations reported a relation or possible relation. For example, Nuevo et al [51] found a potential link between ReHub, an interactive telerehabilitation system, and a fall. However, no studies reported a serious adverse event that was related to telerehabilitation. A summary of the incidence, severity, and relatedness of adverse events across various rehabilitation populations is illustrated in Table 2 and Figure 2.

**Table 2.** Incidence of adverse events per 100 sessions across rehabilitation populations.

Rehabilitation population	Number of adverse events per 100 sessions
Cardiac	0.28
Musculoskeletal	0.05
Neurological	0.58
Pulmonary	0.00
Stroke	0.61
Other	0.00

Regarding the control groups, a total of 137 adverse events were recorded during 29,688 sessions (0.46 adverse events per 100 sessions). As with the intervention groups, information on the type, severity, and relatedness of adverse events was infrequently provided. Among the 12 (32%) studies that documented adverse events, 9 offered additional details. Most of these adverse events were mild or nonserious, including diaphoresis [33], flu symptoms [41], and pain [41,57]. One study by van der Kolk et al [57], which provided asynchronous telerehabilitation to 23 patients with Parkinson disease, reported 4 serious adverse events: supraventricular tachycardia, hip fracture, fall-related injury, and severe dyskinesia. These events were determined to be unrelated to the control group protocol. Conversely, the same study also identified 4 nonserious adverse events related to back pain, which were found to be potentially related to the control group protocol.

### Risk of Bias Assessment

**Figure 3.**
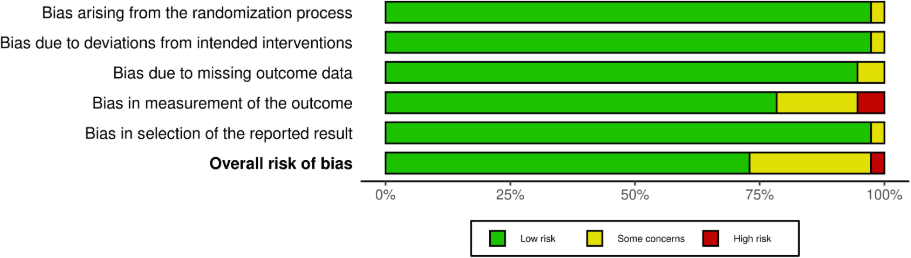
Risk of bias assessment. Red denotes high risk, yellow unclear risk, and green low risk.

The risk of bias assessment is detailed in Figure 3. In total, 36 (97%) studies demonstrated adequate randomization and allocation concealment, although 1 study [28] did not meet the criteria due to an insufficient description of the randomization process (Multimedia Appendix 4) [22-32,34-59]. Regarding blinding, it is often impractical to blind participants and personnel delivering the intervention in telerehabilitation studies [9]. However, 6 (16%) studies [31,41,47-50] successfully blinded trial personnel to participants’ group assignments. In 31 (84%) studies, the absence of blinding did not lead to deviations from the intended interventions. Potential bias was identified in 2 (5%) studies [29,45], where deviations were mainly attributed to missing information. With respect to the availability of outcome data, 34 (92%) studies were rated as low risk. Regarding outcome measurement, 9 (24%) studies [28,33,34,39,44-46,52,55] had potential bias due to either outcome assessors being aware of participant assignments or missing information. In total, 36 (97%) studies had no risk of bias related to the selection of reported results. Overall, 26 (70%) studies were assessed as having a low risk of bias [22-27,29,30,32,34-38,40-43,47-51,54,57,58], 9 (24%) had a moderate risk of bias [31,33,39,44-46,53,55,56], and 2 (5%) had a high risk of bias [28,52].

### Certainty of Evidence Evaluation

For the adverse event outcome, there was serious inconsistency in the results; however, the risk of bias, indirectness of evidence, and imprecision of results were not considered serious. Overall, this led to a moderate certainty of evidence.

## Discussion

### Principal Findings

The objective of this systematic review was to determine the association between telerehabilitation and adverse events reported in randomized controlled trials. These findings build on a recent scoping review by Yau et al [16] and provide a more in-depth analysis of adverse events and study rigor. To our knowledge, this is the first systematic review to provide a comprehensive summary of adverse events in telerehabilitation across numerous patient populations. Overall, 68% (n=25) of the included studies reported no adverse events in either their telerehabilitation interventions or control groups. Among the studies that did report adverse events, the majority were classified as nonserious or mild and physical in nature. With respect to safety and monitoring, 59% (n=22) of studies used tools such as smartwatches and web-based software to allow digital tracking and monitoring by rehabilitation personnel. Additionally, 54% (n=20) of studies incorporated further risk-mitigation strategies, such as patient education sessions and adjustments to activities for safety within the home environment.

### Comparison to Prior Work

When comparing these findings to in-person rehabilitation, outcomes vary depending on the type of rehabilitation and patient populations involved. The most frequent form of telerehabilitation in this review was musculoskeletal-related, including strengthening, stretching, and balance exercises. Niemeijer et al [60] conducted a systematic review and meta-analysis of 773 studies that evaluated the relative risk of adverse events for musculoskeletal rehabilitation in various in-person settings, including outpatient centers, hospitals, and long-term care homes. Compared to control groups, in-person rehabilitation did not increase the risk of serious adverse events. However, there was a relative risk of 1.19 (95% CI 1.09-1.30) for nonserious adverse events, translating to a number needed to harm of 6. With respect to cardiac rehabilitation, Dalal et al [61] conducted a systematic review and meta-analysis of 23 randomized controlled trials comparing center-based and home-based cardiac rehabilitation. They found no significant differences in outcomes such as mortality and cardiac events. Other systematic reviews on in-person pulmonary rehabilitation in patients with chronic obstructive pulmonary disease [62] and burn rehabilitation [63] reported comparable findings, including a generally low incidence of adverse events, most of which were mild or nonserious. Therefore, across various patient populations, telerehabilitation appears to have a safety profile similar to that of in-person rehabilitation. Although adverse events are generally infrequent, mild or nonserious incidents cannot be entirely excluded.

The majority of reported adverse events in this systematic review were classified as physical in nature, with 111 of the 201 adverse events falling into this category. The most frequently reported physical adverse events were “falls” and “pain.” These findings align with a systematic review of clinical outcomes in telerehabilitation for individuals with physical disabilities, which also identified “falls” as a common adverse event across 22 studies [64]. This prevalence may stem from the clinical conditions or baseline risk factors of individuals requiring rehabilitation, which heighten susceptibility to incidents like falls. Therefore, regular check-ins focused specifically on pain management and fall prevention strategies may be particularly beneficial in telerehabilitation. It is also important to note that the definition of a “physical” adverse event varied significantly among studies, with some including “fatigue” and “tiredness” within this classification. These symptoms are expected following participation in therapy and exercise and may not be consistently classified as adverse events by investigators across all studies. Future research should establish standardized definitions for adverse events in telerehabilitation to enhance comparability across studies.

### Implications of Findings

Adverse events also differed among studies that featured real-time interactions between patients and their therapists compared to those using asynchronous methods, during which therapy and feedback occurred at different times. Specifically, 45 adverse events across 2972 sessions (1.52 per 100 sessions) were reported in synchronous telerehabilitation compared to 156 adverse events across 37,960 sessions (0.41 per 100 sessions) in asynchronous telerehabilitation. This difference may be attributed to the more immediate and direct observation of adverse events in synchronous interventions, where therapists can witness and report events in real time. Additionally, real-time interactions in synchronous sessions may encourage patients to be more active or engage in higher-intensity activities, potentially leading to more frequent adverse events. In contrast, some asynchronous interventions, such as the cardiac telerehabilitation program by Snoek et al [53], relied on scheduled check-ins where patients self-reported adverse events. Other asynchronous interventions, such as a strengthening intervention by Hume et al [37], allowed patients to record adverse events in a notebook at any time. Future research is needed to better understand how the mode of communication in telerehabilitation influences the reporting and recognition of adverse events.

An important consideration in evaluating adverse events is whether they are directly attributable to the intervention or incidental to the patient’s underlying conditions. Of the 12 (32%) interventions reporting adverse events, 9 provided information on their relatedness to the intervention: 1 was deemed related, 3 had mixed causality, and 5 were unrelated. For instance, Keteyian et al [28] reported a fall related to their remote cardiac rehabilitation program, although they did not explore which specific aspects of the intervention may have contributed to this event. van der Kolk et al [57] found that 74% of adverse events in their aerobic exercise telerehabilitation program for patients with Parkinson disease were unrelated to the intervention, though a potential relationship could not be excluded in the remaining cases, as these events may have represented a worsening or recurrence of the patient’s pre-existing conditions. Notably, all potentially related adverse events were classified as nonserious. Further research is needed to clarify which specific components of telerehabilitation interventions may account for the varying relationships observed between the interventions and adverse events. Moreover, standardized reporting frameworks should be implemented in future studies to capture all relevant characteristics of adverse events, including their relatedness and severity.

The included studies used a range of approaches to deliver telerehabilitation, from various technologies to more traditional methods like booklet-based exercise programs. Studies using videoconferencing reported the highest rate of adverse events, with 1.96 events per 100 sessions. This may be due to the real-time nature of videoconferencing, which could lead to increased reporting from therapists compared to methods that rely on patient self-report. The second-highest rate was observed in studies where patients followed independent exercise programs (1.23 events per 100 sessions), possibly due to reduced therapist monitoring of safety. Studies involving technologies such as virtual reality and exergaming software had 0.77 events per 100 sessions, though they were only used in 2 (5%) studies, likely because they are newer technologies. In contrast, studies that implemented monitoring devices (eg, heart rate monitors, electrocardiograms, and motion sensors) and uploaded their data to a mobile or web platform for review by rehabilitation personnel had the lowest rate of adverse events (0.31 events per 100 sessions). The abundance of quantitative data these devices provided may have facilitated more thorough monitoring and detection of potential issues, allowing for timely intervention. These findings suggest a potential benefit in using telerehabilitation methods with greater therapist oversight or continuous data monitoring, though further research is needed to fully understand these relationships, especially given the novelty of some technologies.

A significant aspect of reducing adverse events in telerehabilitation is the implementation of safety measures, and 32 (86%) studies provided practical examples of how these were integrated into their interventions. For instance, in a pulmonary telerehabilitation program for survivors of COVID-19, Capin et al [29] required physiotherapists to complete a systematic safety checklist before each session, which included reviewing participants’ emergency contact information, vital signs, and any adverse events (eg, emergency room visits, hospitalizations, and new or worsening symptoms) since the last visit. Similarly, Peng et al [24] conducted a 60-minute education session on exercise before rehabilitation and requested that patients’ caregivers be present during the sessions. Notably, both of these studies reported 0 adverse events across 1904 telerehabilitation sessions. Among the 11 (30%) studies that implemented scheduled safety check-ins with rehabilitation personnel, only 2 recorded adverse events [53,58]. Additionally, 5 (13%) studies required the on-call availability of personnel, with just 2 reporting any adverse events [39,52]. These findings suggest a potential association between the implementation of defined safety strategies and a reduced number of adverse events during telerehabilitation. However, more research is needed to objectively evaluate the efficacy of these measures. Professional organizations, including the American Physical Therapy Association, have emphasized the need for further research into the effectiveness of specific safety protocols for telerehabilitation, such as standardized assessments and checklists [9]. This underscores the importance of developing and continuously updating evidence-based guidelines to ensure patient safety in telerehabilitation programs as they continue to expand in use.

### Strengths and Limitations

The strengths of this study stem from a comprehensive search strategy created in collaboration with a health sciences librarian. The review process adhered to PRISMA best practice guidelines, with all phases, including data screening and extraction, conducted independently and in duplicate by 2 investigators (HS and JC). Additionally, an analysis of study quality using the Cochrane risk of bias 2 tool showed that 70% (n=26) of the included studies had a low risk of bias.

However, this study also has limitations. First, 76% (n=28) of included studies provided insufficient reporting on adverse events, particularly regarding severity and relatedness. This limitation may have hindered our ability to capture a comprehensive understanding of adverse events in telerehabilitation settings. This issue has been well-documented in other reviews [59,65,66]. Therefore, it is essential to emphasize the importance of thorough data collection on adverse events and to develop standardized reporting frameworks. Second, there was a small sample size in some studies, with 5 studies having intervention groups of fewer than 10 participants. The limited statistical power of these studies may have reduced their ability to detect significant differences in adverse event incidence between the intervention and control groups. Future research should prioritize larger, well-powered randomized controlled trials to improve the reliability of findings and allow for more robust conclusions regarding the safety of telerehabilitation. Third, there was an underrepresentation of certain telerehabilitation settings (eg, burn rehabilitation and cancer rehabilitation) as well as younger and older age groups. Notably, only 12 (32%) studies had a mean participant aged 65 years and older, and none had a mean age younger than 18 years. This may limit the generalizability of the findings to specific telerehabilitation settings and age groups. Efforts were made to mitigate this limitation by including telerehabilitation interventions across a range of patient populations. Broadening the search criteria to explore databases specializing in pediatric or geriatric populations may enhance the applicability of the results. Fourth, the participants in randomized controlled trials often represent “ideal” patients without complex health profiles or comorbidities, limiting the applicability of our findings to the general public. This may lead to an overestimation of the safety and effectiveness of telerehabilitation. Fifth, this study did not include a meta-analysis, which may have limited the ability to draw more precise conclusions regarding the safety of telerehabilitation. Future systematic reviews should include meta-analyses to better quantify the incidence, type, severity, and relatedness of adverse events associated with telerehabilitation. Sixth, only studies written in English were included, which may have led to language bias and the exclusion of relevant studies published in other languages. To address this, future research could expand the inclusion criteria to allow for non-English studies or use translation services to capture a broader range of evidence.

### Conclusions

This systematic review examining adverse events in telerehabilitation found that telerehabilitation can be a safe alternative to in-person rehabilitation. A total of 37 studies were included, with 25 (68%) reporting 0 adverse events across 24,450 sessions. Among those that did report adverse events, most were physical, nonserious, and unrelated to the telerehabilitation intervention. Additionally, the included studies identified several safety practices that may enhance telerehabilitation delivery. However, further research is needed to determine the impact of specific protocols on telerehabilitation safety. Future studies should also prioritize the development of standardized reporting for adverse events to address the underreporting observed in current randomized controlled trials.

## Supplementary material

10.2196/68681 Multimedia Appendix 1 Search strategy.

10.2196/68681 Multimedia Appendix 2 Participant and intervention characteristics.

10.2196/68681 Multimedia Appendix 3 Adverse events in the intervention and control groups.

10.2196/68681 Multimedia Appendix 4 Risk of bias assessment of individual studies.

10.2196/68681 Checklist 1 PRISMA (Preferred Reporting Items for Systematic Reviews and Meta-Analyses) checklist.

## Abbreviations

PRISMA: Preferred Reporting Items for Systematic Reviews and Meta-Analyses
TIDieR: Template for Intervention Description and Replication
