# Supplementary material for: The Safety of Telerehabilitation: Systematic Review
Source: JMIR Rehabil Assist Technol. 2025 Jul 9;12:e68681. doi: 10.2196/68681 (PMC12266302; doi:10.2196/68681)
Supplement: Multimedia Appendix 1 [file rehab-v12-e68681-s001.docx]

**PRISMA Initial Results:** total number of results before duplicates removed

| **Database [Platform]** Searches run January 8, 2024. *Results limited to* *June 22, 2023 to current, where possible.* | **Results** |
| --- | --- |
| MEDLINE(R) ALL June 22, 2023 to January 05, 2024 [Ovid] | 160 |
| Embase Classic+Embase June 22, 2023 to 2024 January 05 [Ovid] | 209 |
| EBM Reviews - Cochrane Central Register of Controlled Trials December 2023 [Ovid | 240 |
| APA PsycInfo 2023 to January Week 1 2024 [Ovid] | 11 |
| CINAHL June 22, 2023 to current [EBSCOHost] | 47 |
| **TOTAL** | **667** |

**MEDLINE(R) ALL**1946 to January 05, 2024
Search Strategy:

| **#** | **Searches** | **Results** |
| --- | --- | --- |
| 1 | Telemedicine/ | 38676 |
| 2 | Videoconferencing/ | 2351 |
| 3 | Remote Consultation/ | 5799 |
| 4 | or/1-3 | 44371 |
| 5 | exp Rehabilitation/ | 358152 |
| 6 | 4 and 5 | 3243 |
| 7 | rehab*.tw,kf. | 230570 |
| 8 | 4 and 7 | 1281 |
| 9 | rh.fs. | 208931 |
| 10 | 4 and 9 | 962 |
| 11 | (remote* or tele* or virtual* or "vr" or videoconferenc* or "video conferenc*").tw,kf. | 488967 |
| 12 | (5 or 7) and 11 | 19291 |
| 13 | or/6,8,10,12 | 21156 |
| 14 | Telerehabilitation/ | 1078 |
| 15 | telerehab*.tw,kf. | 2218 |
| 16 | "e-rehab*".tw,kf. | 26 |
| 17 | or/13-16 | 21616 |
| 18 | Safety/ | 42067 |
| 19 | Patient Safety/ | 25793 |
| 20 | (safe or safety or safeties or safely or unsafe).tw,kf. | 1151653 |
| 21 | Patient Harm/ | 228 |
| 22 | (harm or harmed or harmful or harming or harms).tw,kf. | 175026 |
| 23 | (risk or risks).tw,kf. | 3002052 |
| 24 | Medical Errors/ | 17800 |
| 25 | (adverse* adj5 (effect* or event* or incident or incidents or outcome*)).tw,kf. | 579791 |
| 26 | (critical adj5 (event* or incident or incidents or outcome*)).tw,kf. | 23390 |
| 27 | (negativ* adj5 (effect* or event* or impact* or incident or incidents or mistake or mistakes or outcome*)).tw,kf. | 221884 |
| 28 | (therap* adj5 (accident* or error or errors or excessive or incident or incidents or mistake or mistakes or unnecessary or wrong)).tw,kf. | 7501 |
| 29 | (outcome* adj5 harm*).tw,kf. | 3115 |
| 30 | complication*.tw,kf. | 1226429 |
| 31 | ae.fs. | 2025167 |
| 32 | or/18-31 | 6412119 |
| 33 | 17 and 32 | 5546 |
| 34 | limit 33 to english language | 5360 |
| 35 | limit 34 to yr="2013 -Current" | 4184 |
| 36 | limit 34 to ed=20230622-20240105 | 239 |
| 37 | ("20230622*" or "20230623* or 20230624*" or "20230625* or 20230626*" or "20230627* or 20230628*" or "20230629* or 20230630*" or "202307*" or "202308*" or "202309*" or "2023010*" or "2023011*" or "2023012*" or "202401*").dt. | 541326 |
| 38 | 37 and 34 | 209 |
| 39 | 36 or 38 | 356 |
| 40 | (Randomized Controlled Trial or Controlled Clinical Trial or Pragmatic Clinical Trial or Equivalence Trial or Clinical Trial, Phase III).pt. | 701870 |
| 41 | Randomized Controlled Trial/ | 606173 |
| 42 | exp Randomized Controlled Trials as Topic/ | 169901 |
| 43 | "Randomized Controlled Trial (topic)"/ | 0 |
| 44 | Controlled Clinical Trial/ | 95517 |
| 45 | exp Controlled Clinical Trials as Topic/ | 175616 |
| 46 | "Controlled Clinical Trial (topic)"/ | 0 |
| 47 | Randomization/ | 107058 |
| 48 | Random Allocation/ | 107058 |
| 49 | Double-Blind Method/ | 177106 |
| 50 | Double Blind Procedure/ | 0 |
| 51 | Double-Blind Studies/ | 177106 |
| 52 | Single-Blind Method/ | 33149 |
| 53 | Single Blind Procedure/ | 0 |
| 54 | Single-Blind Studies/ | 33149 |
| 55 | Placebos/ | 35934 |
| 56 | Placebo/ | 0 |
| 57 | Control Groups/ | 2070 |
| 58 | Control Group/ | 2070 |
| 59 | (random* or sham or placebo*).ti,ab,hw,kf. | 1868535 |
| 60 | ((singl* or doubl*) adj (blind* or dumm* or mask*)).ti,ab,hw,kf. | 272148 |
| 61 | ((tripl* or trebl*) adj (blind* or dumm* or mask*)).ti,ab,hw,kf. | 1735 |
| 62 | (control* adj3 (study or studies or trial* or group*)).ti,ab,kf. | 1267900 |
| 63 | (Nonrandom* or non random* or non-random* or quasi-random* or quasirandom*).ti,ab,hw,kf. | 56637 |
| 64 | allocated.ti,ab,hw. | 86904 |
| 65 | ((open label or open-label) adj5 (study or studies or trial*)).ti,ab,hw,kf. | 46565 |
| 66 | ((equivalence or superiority or non-inferiority or noninferiority) adj3 (study or studies or trial*)).ti,ab,hw,kf. | 12822 |
| 67 | (pragmatic study or pragmatic studies).ti,ab,hw,kf. | 635 |
| 68 | ((pragmatic or practical) adj3 trial*).ti,ab,hw,kf. | 8091 |
| 69 | ((quasiexperimental or quasi-experimental) adj3 (study or studies or trial*)).ti,ab,hw,kf. | 13120 |
| 70 | (phase adj3 (III or "3") adj3 (study or studies or trial*)).ti,hw,kf. | 36092 |
| 71 | or/40-70 [Citation: RCT / CCT - MEDLINE, Embase. In: CADTH Search Filters Database. Ottawa: CADTH; 2024: https://searchfilters.cadth.ca/link/122. Accessed 2024-01-05. ] | 2677178 |
| 72 | 39 and 71 | 160 |

**Embase Classic+Embase**1947 to 2024 January 05
Search Strategy:

| **#** | **Searches** | **Results** |
| --- | --- | --- |
| 1 | telemedicine/ | 46334 |
| 2 | videoconferencing/ | 9469 |
| 3 | teleconsultation/ | 15897 |
| 4 | or/1-3 | 66202 |
| 5 | exp rehabilitation/ | 521707 |
| 6 | 4 and 5 | 3426 |
| 7 | rehab*.tw,kw. | 337131 |
| 8 | 4 and 7 | 2219 |
| 9 | rh.fs. | 178839 |
| 10 | 4 and 9 | 979 |
| 11 | (remote* or tele* or virtual* or "vr" or videoconferenc* or "video conferenc*").tw,kw. | 634159 |
| 12 | (5 or 7 or 9) and 11 | 29886 |
| 13 | or/6,8,10,12 | 30936 |
| 14 | telerehabilitation/ | 2824 |
| 15 | telerehab*.tw,kw. | 2515 |
| 16 | "e-rehab*".tw,kw. | 39 |
| 17 | or/13-16 | 31406 |
| 18 | patient safety/ | 156141 |
| 19 | (safe or safety or safeties or safely or unsafe).tw,kw. | 1733669 |
| 20 | patient harm/ | 2998 |
| 21 | (harm or harmed or harmful or harming or harms).tw,kw. | 224630 |
| 22 | patient risk/ | 11808 |
| 23 | (risk or risks).tw,kw. | 4334889 |
| 24 | medical error/ | 21076 |
| 25 | (adverse* adj5 (effect* or event* or incident or incidents or outcome*)).tw,kw. | 896665 |
| 26 | (critical adj5 (event* or incident or incidents or outcome*)).tw,kw. | 31353 |
| 27 | (negativ* adj5 (effect* or event* or impact* or incident or incidents or mistake or mistakes or outcome*)).tw,kw. | 289555 |
| 28 | (therap* adj5 (accident* or error or errors or excessive or incident or incidents or mistake or mistakes or unnecessary or wrong)).tw,kw. | 12061 |
| 29 | (outcome* adj5 harm*).tw,kw. | 4039 |
| 30 | complication*.tw,kw. | 1780214 |
| 31 | ae.fs. | 1457809 |
| 32 | or/18-31 | 8412532 |
| 33 | 17 and 32 | 8722 |
| 34 | limit 33 to english language | 8458 |
| 35 | limit 34 to (books or chapter or conference abstract or conference paper or "conference review") | 2414 |
| 36 | 33 not 35 | 6308 |
| 37 | limit 36 to dc=20230622-20240105 | 499 |
| 38 | (Randomized Controlled Trial or Controlled Clinical Trial or Pragmatic Clinical Trial or Equivalence Trial or Clinical Trial, Phase III).pt. | 0 |
| 39 | Randomized Controlled Trial/ | 803999 |
| 40 | exp Randomized Controlled Trials as Topic/ | 267774 |
| 41 | "Randomized Controlled Trial (topic)"/ | 267649 |
| 42 | Controlled Clinical Trial/ | 472303 |
| 43 | exp Controlled Clinical Trials as Topic/ | 277219 |
| 44 | "Controlled Clinical Trial (topic)"/ | 13471 |
| 45 | Randomization/ | 99284 |
| 46 | Random Allocation/ | 93968 |
| 47 | Double-Blind Method/ | 192163 |
| 48 | Double Blind Procedure/ | 217163 |
| 49 | Double-Blind Studies/ | 174192 |
| 50 | Single-Blind Method/ | 51049 |
| 51 | Single Blind Procedure/ | 53117 |
| 52 | Single-Blind Studies/ | 53117 |
| 53 | Placebos/ | 361274 |
| 54 | Placebo/ | 418222 |
| 55 | Control Groups/ | 123614 |
| 56 | Control Group/ | 123614 |
| 57 | (random* or sham or placebo*).ti,ab,hw,kf. | 2637590 |
| 58 | ((singl* or doubl*) adj (blind* or dumm* or mask*)).ti,ab,hw,kf. | 374341 |
| 59 | ((tripl* or trebl*) adj (blind* or dumm* or mask*)).ti,ab,hw,kf. | 2288 |
| 60 | (control* adj3 (study or studies or trial* or group*)).ti,ab,kf. | 1789293 |
| 61 | (Nonrandom* or non random* or non-random* or quasi-random* or quasirandom*).ti,ab,hw,kf. | 72467 |
| 62 | allocated.ti,ab,hw. | 112654 |
| 63 | ((open label or open-label) adj5 (study or studies or trial*)).ti,ab,hw,kf. | 89375 |
| 64 | ((equivalence or superiority or non-inferiority or noninferiority) adj3 (study or studies or trial*)).ti,ab,hw,kf. | 19112 |
| 65 | (pragmatic study or pragmatic studies).ti,ab,hw,kf. | 979 |
| 66 | ((pragmatic or practical) adj3 trial*).ti,ab,hw,kf. | 9136 |
| 67 | ((quasiexperimental or quasi-experimental) adj3 (study or studies or trial*)).ti,ab,hw,kf. | 20598 |
| 68 | (phase adj3 (III or "3") adj3 (study or studies or trial*)).ti,hw,kf. | 132373 |
| 69 | or/38-68 [Citation: RCT / CCT - MEDLINE, Embase. In: CADTH Search Filters Database. Ottawa: CADTH; 2024: https://searchfilters.cadth.ca/link/122. Accessed 2024-01-05. ] | 3907038 |
| 70 | 37 and 69 | 207 |

**EBM Reviews - Cochrane Central Register of Controlled Trials**December 2023
Search Strategy:

| **#** | **Searches** | **Results** |
| --- | --- | --- |
| 1 | telemedicine/ | 3602 |
| 2 | videoconferencing/ | 332 |
| 3 | remote consultation/ | 416 |
| 4 | or/1-3 | 4153 |
| 5 | exp Rehabilitation/ | 51034 |
| 6 | 4 and 5 | 768 |
| 7 | rehab*.tw,kw. | 51811 |
| 8 | 4 and 7 | 207 |
| 9 | rh.fs. | 21689 |
| 10 | 4 and 9 | 243 |
| 11 | (remote* or tele* or virtual* or "vr" or videoconferenc* or "video conferenc*").tw,kw. | 59946 |
| 12 | (5 or 7 or 9) and 11 | 7445 |
| 13 | or/6,8,10,12 | 7757 |
| 14 | Telerehabilitation/ | 288 |
| 15 | telerehab*.tw,kw. | 1198 |
| 16 | "e-rehab*".tw,kw. | 9 |
| 17 | or/13-16 | 8111 |
| 18 | safety/ | 16736 |
| 19 | patient safety/ | 1524 |
| 20 | (safe or safety or safeties or safely or unsafe).tw,kw. | 338369 |
| 21 | patient harm/ | 15 |
| 22 | (harm or harmed or harming or harms).tw,kw. | 7989 |
| 23 | (risk or risks).tw,kw. | 279398 |
| 24 | Medical Errors/ | 190 |
| 25 | (adverse* adj5 (effect* or event* or incident or incidents or outcome*)).tw,kw. | 200718 |
| 26 | (critical adj5 (event* or incident or incidents or outcome*)).tw,kw. | 1289 |
| 27 | (negativ* adj5 (effect* or event* or impact* or incident or incidents or mistake or mistakes or outcome*)).tw,kw. | 20160 |
| 28 | (therap* adj5 (accident* or error or errors or excessive or incident or incidents or mistake or mistakes or unnecessary or wrong)).tw,kw. | 3322 |
| 29 | (outcome* adj5 harm*).tw,kw. | 544 |
| 30 | complication*.tw,kw. | 141101 |
| 31 | ae.fs. | 158860 |
| 32 | or/18-31 | 798513 |
| 33 | 17 and 32 | 2603 |
| 34 | limit 33 to english language | 2580 |
| 35 | limit 34 to (conference proceeding or dissertation thesis) | 281 |
| 36 | 34 not 35 | 2299 |
| 37 | limit 36 to yr="2023 -Current" | 240 |

**APA PsycInfo**1806 to January Week 1 2024
Search Strategy:

| **#** | **Searches** | **Results** |
| --- | --- | --- |
| 1 | telemedicine/ | 8624 |
| 2 | videoconferencing/ | 971 |
| 3 | teleconsultation/ | 172 |
| 4 | or/1-3 | 9384 |
| 5 | rehabilitation/ | 23506 |
| 6 | 4 and 5 | 211 |
| 7 | rehab*.tw,id. | 73510 |
| 8 | 4 and 7 | 359 |
| 9 | (remote* or tele* or virtual* or "vr" or videoconferenc* or "video conferenc*").tw,id. | 133520 |
| 10 | (5 or 7) and 9 | 3644 |
| 11 | or/6,8,10 | 3669 |
| 12 | telerehabilitation/ | 273 |
| 13 | telerehab*.tw,id. | 357 |
| 14 | "e-rehab*".tw,id. | 10 |
| 15 | or/11-14 | 3774 |
| 16 | safety/ | 17834 |
| 17 | patient safety/ | 3771 |
| 18 | (safe or safety or safeties or safely or unsafe).tw,id. | 121489 |
| 19 | (harm or harmed or harming or harms).tw,id. | 48485 |
| 20 | (risk or risks).tw,id. | 483377 |
| 21 | (adverse* adj5 (effect* or event* or incident or incidents or outcome*)).tw,id. | 50576 |
| 22 | (critical adj5 (event* or incident or incidents or outcome*)).tw,id. | 8336 |
| 23 | (negativ* adj5 (effect* or event* or impact* or incident or incidents or mistake or mistakes or outcome*)).tw,id. | 98051 |
| 24 | (therap* adj5 (accident* or error or errors or excessive or incident or incidents or mistake or mistakes or unnecessary or wrong)).tw,id. | 1303 |
| 25 | (outcome* adj5 harm*).tw,id. | 1161 |
| 26 | complication*.tw,id. | 28462 |
| 27 | or/16-26 | 735054 |
| 28 | 15 and 27 | 595 |
| 29 | limit 28 to english language | 566 |
| 30 | limit 29 to (bibliography or chapter or dissertation or review-book or review-media or review-software & other) | 82 |
| 31 | 28 not 30 | 513 |
| 32 | limit 31 to yr="2023 -Current" | 32 |
| 33 | Experiment Controls/ or Placebo/ or Randomized Controlled Trials/ or Randomized Clinical Trials/ | 8952 |
| 34 | (random* or sham or placebo*).ti,ab,id,hw,mf. | 290908 |
| 35 | ((singl* or doubl*) adj (blind* or dumm* or mask*)).ti,ab,id,hw,mf. | 37324 |
| 36 | ((tripl* or trebl*) adj (blind* or dumm* or mask*)).ti,ab,id,hw,mf. | 124 |
| 37 | (control* adj3 (study or studies or trial* or group*)).ti,ab,id. | 202512 |
| 38 | (nonrandom* or non random* or non-random* or quasi-random* or quasirandom*).ti,ab,id,hw,mf. | 6716 |
| 39 | allocated.ti,ab,hw,mf. | 13270 |
| 40 | ((open label or open-label) adj5 (study or studies or trial*)).ti,ab,id,hw,mf. | 5350 |
| 41 | ((equivalence or superiority or non-inferiority or noninferiority) adj3 (study or studies or trial*)).ti,ab,id,hw,mf. | 1240 |
| 42 | (pragmatic study or pragmatic studies).ti,ab,id,hw,mf. | 154 |
| 43 | ((pragmatic or practical) adj3 trial*).ti,ab,id,hw,mf. | 1176 |
| 44 | ((quasiexperimental or quasi-experimental) adj3 (study or studies or trial*)).ti,ab,id,hw,mf. | 7340 |
| 45 | (phase adj3 (III or "3") adj3 (study or studies or trial*)).ti,id,hw,mf. | 532 |
| 46 | or/33-45 | 420834 |
| 47 | 32 and 46 | 11 |

**CINAHL**

Search Strategy:

| **#** | **Query** | **Limiters/Expanders** | **Results** |
| --- | --- | --- | --- |
| S1 | (MH "Telemedicine") | Expanders - Apply equivalent subjects Search modes - Boolean/Phrase | 16,596 |
| S2 | (MH "Videoconferencing") | Expanders - Apply equivalent subjects Search modes - Boolean/Phrase | 3,316 |
| S3 | (MH "Remote Consultation") | Expanders - Apply equivalent subjects Search modes - Boolean/Phrase | 3,260 |
| S4 | S1 OR S2 OR S3 | Expanders - Apply equivalent subjects Search modes - Boolean/Phrase | 21,685 |
| S5 | (MH "Rehabilitation+") | Expanders - Apply equivalent subjects Search modes - Boolean/Phrase | 343,013 |
| S6 | S4 AND S5 | Expanders - Apply equivalent subjects Search modes - Boolean/Phrase | 1,206 |
| S7 | TI (rehab*) OR AB (rehab*) | Expanders - Apply equivalent subjects Search modes - Boolean/Phrase | 117,766 |
| S8 | S4 AND S7 | Expanders - Apply equivalent subjects Search modes - Boolean/Phrase | 587 |
| S9 | MJ Rehabilitation | Expanders - Apply equivalent subjects Search modes - Boolean/Phrase | 121,064 |
| S10 | S4 AND S9 | Expanders - Apply equivalent subjects Search modes - Boolean/Phrase | 669 |
| S11 | S5 OR S7 | Expanders - Apply equivalent subjects Search modes - Boolean/Phrase | 411,523 |
| S12 | TI (remote* or tele* or virtual* or "vr" or videoconferenc* or "video conferenc*") OR AB (remote* or tele* or virtual* or "vr" or videoconferenc* or "video conferenc*") | Expanders - Apply equivalent subjects Search modes - Boolean/Phrase | 142,671 |
| S13 | S11 AND S12 | Expanders - Apply equivalent subjects Search modes - Boolean/Phrase | 11,897 |
| S14 | (MH "Telerehabilitation") | Expanders - Apply equivalent subjects Search modes - Boolean/Phrase | 833 |
| S15 | TI telerehab* OR AB telerehab* | Expanders - Apply equivalent subjects Search modes - Boolean/Phrase | 929 |
| S16 | TI ("e-rehab*") OR AB ("e-rehab*") | Expanders - Apply equivalent subjects Search modes - Boolean/Phrase | 15 |
| S17 | S6 OR S8 OR S10 OR S13 OR S14 OR S15 OR S16 | Expanders - Apply equivalent subjects Search modes - Boolean/Phrase | 12,743 |
| S18 | (MH "Safety") | Expanders - Apply equivalent subjects Search modes - Boolean/Phrase | 31,308 |
| S19 | (MH "Patient Safety") | Expanders - Apply equivalent subjects Search modes - Boolean/Phrase | 78,295 |
| S20 | TI (safe or safety or safeties or safely or unsafe or harm or harmed or harmful or harming or harms or risk or risks) OR AB (safe or safety or safeties or safely or unsafe or harm or harmed or harmful or harming or harms or risk or risks) | Expanders - Apply equivalent subjects Search modes - Boolean/Phrase | 1,243,644 |
| S21 | (MH "Health Care Errors") OR (MH "Treatment Errors") | Expanders - Apply equivalent subjects Search modes - Boolean/Phrase | 13,676 |
| S22 | TI (adverse* N4 (effect* or event* or incident or incidents or outcome*)) OR AB (adverse* N4 (effect* or event* or incident or incidents or outcome*)) | Expanders - Apply equivalent subjects Search modes - Boolean/Phrase | 161,507 |
| S23 | TI (critical N5 (event* or incident or incidents or outcome*)) OR AB (critical N5 (event* or incident or incidents or outcome*)) | Expanders - Apply equivalent subjects Search modes - Boolean/Phrase | 8,457 |
| S24 | TI (negativ* adj4 (effect* or event* or impact* or incident or incidents or mistake or mistakes or outcome*)) OR AB (negativ* adj4 (effect* or event* or impact* or incident or incidents or mistake or mistakes or outcome*)) | Expanders - Apply equivalent subjects Search modes - SmartText Searching | 933 |
| S25 | TI (therap* N5 (accident* or error or errors or excessive or incident or incidents or mistake or mistakes or unnecessary or wrong)) OR AB (therap* N5 (accident* or error or errors or excessive or incident or incidents or mistake or mistakes or unnecessary or wrong)) | Expanders - Apply equivalent subjects Search modes - Boolean/Phrase | 2,128 |
| S26 | TI (outcome* N5 harm*) OR AB (outcome* N5 harm*) | Expanders - Apply equivalent subjects Search modes - Boolean/Phrase | 1,612 |
| S27 | TI complication* OR AB complication* | Expanders - Apply equivalent subjects Search modes - Boolean/Phrase | 243,809 |
| S28 | MJ Adverse Effects | Expanders - Apply equivalent subjects Search modes - Boolean/Phrase | 325,848 |
| S29 | S18 OR S19 OR S20 OR S21 OR S22 OR S23 OR S24 OR S25 OR S26 OR S27 OR S28 | Expanders - Apply equivalent subjects Search modes - Boolean/Phrase | 1,696,115 |
| S30 | S17 AND S29 | Expanders - Apply equivalent subjects Search modes - Boolean/Phrase | 2,473 |
| S31 | S17 AND S29 | Limiters - English Language; Peer Reviewed Expanders - Apply equivalent subjects Search modes - Boolean/Phrase | 2,285 |
| S32 | EM 20230622*- | Limiters - English Language; Peer Reviewed Expanders - Apply equivalent subjects Search modes - Boolean/Phrase | 113,338 |
| S33 | S31 AND S32 | Expanders - Apply equivalent subjects Search modes - Boolean/Phrase | 117 |
| S34 | (MH "Randomized Controlled Trials+") | Expanders - Apply equivalent subjects Search modes - Boolean/Phrase | 142,113 |
| S35 | (MH "Double-Blind Studies") | Expanders - Apply equivalent subjects Search modes - Boolean/Phrase | 54,709 |
| S36 | (MH "Single-Blind Studies") | Expanders - Apply equivalent subjects Search modes - Boolean/Phrase | 16,116 |
| S37 | (MH "Random Assignment") | Expanders - Apply equivalent subjects Search modes - Boolean/Phrase | 83,041 |
| S38 | (MH "Pretest-Posttest Design") | Expanders - Apply equivalent subjects Search modes - Boolean/Phrase | 54,617 |
| S39 | (MH "Cluster Sample") | Expanders - Apply equivalent subjects Search modes - Boolean/Phrase | 5,412 |
| S40 | TI (randomised OR randomized) | Expanders - Apply equivalent subjects Search modes - Boolean/Phrase | 147,505 |
| S41 | AB (random*) | Expanders - Apply equivalent subjects Search modes - Boolean/Phrase | 404,826 |
| S42 | TI (trial) | Expanders - Apply equivalent subjects Search modes - Boolean/Phrase | 189,750 |
| S43 | MH "Sample Size") AND AB (assigned OR allocated OR control) | Expanders - Apply equivalent subjects Search modes - Boolean/Phrase | 4,460 |
| S44 | (MH "Placebos") | Expanders - Apply equivalent subjects Search modes - Boolean/Phrase | 14,308 |
| S45 | PT randomized controlled trial | Expanders - Apply equivalent subjects Search modes - Boolean/Phrase | 155,593 |
| S46 | AB (control W5 group) | Expanders - Apply equivalent subjects Search modes - Boolean/Phrase | 147,586 |
| S47 | (MH "Comparative Studies") | Expanders - Apply equivalent subjects Search modes - Boolean/Phrase | 476,598 |
| S48 | (MH "Crossover Design") | Expanders - Apply equivalent subjects Search modes - Boolean/Phrase | 22,133 |
| S49 | AB (cluster W3 RCT) | Expanders - Apply equivalent subjects Search modes - Boolean/Phrase | 504 |
| S50 | S34 OR S35 OR S36 OR S37 OR S38 OR S39 OR S40 OR S41 OR S42 OR S43 OR S44 OR S45 OR S46 OR S47 OR S48 OR S49 | Expanders - Apply equivalent subjects Search modes - Boolean/Phrase | 1,045,382 |
| S51 | S33 AND S50 | Expanders - Apply equivalent subjects Search modes - Boolean/Phrase | 47 |
