# Supplementary material for: The Safety of Telerehabilitation: Systematic Review
Source: JMIR Rehabil Assist Technol. 2025 Jul 9;12:e68681. doi: 10.2196/68681 (PMC12266302; doi:10.2196/68681)
Supplement: Multimedia Appendix 2 [file rehab-v12-e68681-s002.docx]

| **Study/Year/Country** | **Aim** | **Participants (n; % Female; Mean Age [Years])** | **Intervention Group (IG) and Control Group (CG)** | **Intervention: Hybrid/Full and Synchronous/ Asynchronous Telerehabilitation** | **Intervention: Who Provided and Where** | **Intervention: Frequency and Duration** | **Intervention: Tailoring, Modifications, and Adherence** | **Materials** | **Safety Measures** |
| --- | --- | --- | --- | --- | --- | --- | --- | --- | --- |
| Keteyian et al (2021) USA [28] | To compare exercise training intensity during standard cardiac rehabilitation (CR) versus Hybrid-CR (combined clinic- and remote home-/community-based). | IG: 9 (34.6% female) [63 years] CG: 5 (23.8% female) [58 years] | IG: Cardiac rehab CG: Standard cardiac rehab | Hybrid telerehab Synchronous | **Who provided:** Cardiac rehabilitation staff  **Where:** For in-clinic CR visits: both patient and therapist in clinic. For telerehab sessions: patient at home or community, therapist unknown location | 3 sessions/  week [≥30 min] x 6 weeks | **Tailoring:** Exercise target range was set at 60-80% using individual heart rate (HR) reserve. Exercise intensity was self-titrated using ratings of perceived exertion **Modifications:** Not specified **Adherence:** 91% | - Video application - Chest/wrist device | Not specified |
| Jarbandhan et al (2022) Suriname [45] | To assess feasibility and preliminary effectiveness of a home-based semi-supervised physiotherapy intervention to promote post-stroke mobility in a low resource setting. | IG: 11 (55% female) [61.6 years] CG: 6 (60% female) [62.2 years] | IG: Stroke rehab CG: Usual care | Hybrid telerehab Asynchronous | **Who provided:** Physiotherapist  **Where:** Patient at home, therapist with patient during week 1-4, therapist unknown location during week 5-8 (not patient’s home) | 3 sessions/  week [70 min] x 4 weeks | **Tailoring:** Individually tailored program by physiotherapist **Modifications:** Not specified **Adherence:** 70% | - Pedometer - Watch to measure HR - Hand dynamometer - Questionnaire | Blood pressure (BP) measurements were recorded before, during, and after sessions. |
| Hwang et al (2017) Australia [65] | To determine the efficacy and safety of a short-term, real-time, group-based heart failure rehabilitation program delivered into each participant’s home via an online telerehabilitation system. | IG: 5 (20.8% female) [68 years] CG: 8 (27.6% female) [67 years] | IG: Cardiac rehab CG: Traditional hospital outpatient-based program | Full telerehab Synchronous | **Who provided:** Physiotherapist  **Where:** Patients in their own homes. Therapist in unknown location | 2 sessions/  week [60 min] x 12 weeks | **Tailoring:** Prescription was tailored to the participant’s goal and the treating physiotherapist continuously reviewed it to ensure appropriate progression **Modifications:** Therapist could provide real-time feedback and modification as required  **Adherence:** 83% | - Laptop - Mobile device - Automatic sphygmomanometer - Finger pulse oximeter - Free weights - Resistance bands - Equipment manual - Educational information | Participants self-monitored and reported their BP, HR, and oxygen saturation levels at the start of each session. |
| Hume et al (2022) UK [37] | To assess the feasibility and acceptability of a novel, 12-weeks physical activity tele-coaching intervention in lung transplant recipients. | IG: 3 (42.9% female) [57 years] CG: 1 (20% female) [58 years] | IG: Strengthening and stretching exercises, motivational interviewing CG: Usual care | Full telerehab Asynchronous | **Who provided:** Coach with motivational interviewing experience  **Where:** Patients in their own homes and community. Therapist in unknown location | 7 sessions/  week x 12 weeks | **Tailoring:** Activity goal was set by the app, based on the patient’s physical activity levels (steps/day) in the previous week **Modifications:** Not specified **Adherence:** 82% | - Pedometer - Smartphone app - Linkcare web-based platform - Home exercise booklet - Telephone support | Adverse events were recorded in the adverse event log within the patients’ notes. |
| Gehring et al (2018) Netherlands [54] | To investigate the feasibility of a home-based, remotely guided exercise intervention for patients with gliomas. | IG: 13 (56.5% female) [48 years] CG: 6 (54.5% female) [48 years] | IG: Aerobic training CG: Active lifestyle | Full telerehab Asynchronous | **Who provided:** Physiotherapist  **Where:** Patients in their own homes. Therapist in unknown location | 3 sessions/  week x 24 weeks | **Tailoring:** Individualized exercise prescription. Participants could choose one or more central activities, as long as these could meet the prescribed exercise intensity **Modifications:** Not specified **Adherence:** 79% | - Sports watch - Training experience log - Online platform | The sports watch provided immediate feedback about HR. The physiotherapist monitored the training data on a weekly basis and provided additional feedback by email. |
| Fioratti et al (2022) Brazil [40] | To evaluate the feasibility, usability, and implementation context of a self-management internet-based program based on exercises and pain education (ReabilitaDOR) in people with chronic musculoskeletal pain and to compare this program with a program using only a web-based self-management booklet. | IG: 19 (61.3% female) [40.2 years] CG: 24 (72.7% female) [38.8 years] | IG: Chronic musculoskeletal pain exercise treatment and pain education CG: Web-based self-management booklet | Full telerehab Asynchronous | **Who provided:** Physiotherapists who are specialists in chronic pain treatment  **Where:** Patients in their own homes. Therapist in unknown location | 3 sessions/  week x 8 weeks | **Tailoring:** Not specified **Modifications:** There were health coaching sessions once a week by a physiotherapist which included adaptation of the content of the intervention, if necessary **Adherence:** 90% | - Web-based platforms - Website developed for the study - Videos and animations | The health coach component included encouragement, motivation, coping, revision of instructions and, if necessary, adaptation of the content of the intervention. |
| Donkers et al (2020) Canada [42] | To evaluate adherence to a Web-based, individualized exercise program in moderate-to-severe multiple sclerosis. | IG: 20 (62.5% female) [54.6 years] CG: 11 (68.8% female) [53.8 years] | IG: Neuro rehab CG: Standard physiotherapist- prescribed exercise program | Full telerehab Asynchronous | **Who provided:** Physiotherapist  **Where:** Patients in their own homes. Therapist in unknown location | 2 sessions/  week x 26 weeks | **Tailoring:** Individually prescribed by a physiotherapist at an initial assessment. Programs were individualized in terms of exercises, level of difficulty, and number of sets and repetitions **Modifications:** Every 2 weeks, the treating physiotherapist reviewed participants’ online exercise diary and remotely altered their exercise program as appropriate. Participants were also invited to contact their physiotherapist for a change in their program as needed **Adherence:** 74.8% | - Website containing exercises - Online exercise diary | A multi-disciplinary focus group comprised of 2 patients with advanced disability, 1 physiatrist, and 4 physiotherapists created an additional inventory of exercises for people with disabilities. |
| Cox et al (2022) Australia [34] | To investigate whether home-based telerehabilitation was equivalent to centre-based pulmonary rehabilitation in people with chronic respiratory disease. | IG: 41 (60.3% female) [68 years] CG: 35 (52.2% female) [67 years] | IG: Pulmonary rehab CG: Centre-based pulmonary rehab | Hybrid telerehab Synchronous | **Who provided:** Physiotherapist  **Where:** Patients in their own homes. Therapist in patient’s home for the 1st session, unknown location for remaining sessions | 2 sessions/week [60 min] x 8 weeks | **Tailoring:** Individualized based on symptoms, cardiopulmonary exercise test (CPET), etc.  **Modifications:** Not specified **Adherence:** 81% | - Step-through exercise bike - Tablet computer - Pulse oximeter - Self-management education resources | The pulse oximeter monitored peripheral oxygen saturation and pulse rate during training and at rest. |
| Chen et al (2021)  China [22] | To investigate the effectiveness of telerehabilitation in patients after esophageal cancer surgery during COVID-19. | IG: 11 (27.5% female) [59.6 years] CG: 10 (25% female) [59.8 years] | IG: Pulmonary rehab CG: Standard consultation | Full telerehab Asynchronous | **Who provided:** Medical care group which included the head of the nursing department in thoracic surgery, at least one doctor who participated in the surgery, and three experienced nurses  **Where:** Patients in their own homes. Therapist in unknown location | 12 weeks | **Tailoring:** Not specified **Modifications:** Not specified **Adherence:** Not specified | - Small tips - Training videos - Regular online consulting - WeChat group | Participants received guidance and feedback on current vital signs, wound status, medication, and sleep status via online consulting (WeChat). |
| Capin et al (2022) USA [29] | To determine the safety, feasibility and initial efficacy of a multicomponent telerehabilitation program for COVID-19 survivors. | IG: 13 (46.4% female) [52 years] CG: 5 (38.5% female) [54 years] | IG: High-intensity strength training, aerobic and balance exercises, functional activities, stretching, motivational interviewing CG: Education and weekly check-ins | Full telerehab Synchronous | **Who provided:** Physiotherapist  **Where:** Patients in their own homes. Therapist in unknown location | 1.2 sessions/  week [30 min] x 10 weeks | **Tailoring:** Individual biobehaviourally informed, app-facilitated, multicomponent telerehabilitation sessions **Modifications:** Not specified **Adherence:** 97% | - Zoom - Automated blood pressure cuff - Pulse oximeter - Tablet - Activity monitor - Equipment instruction manual - Ankle weights - Resistance bands - Mobile application | A systematic safety checklist was completed before sessions which included a review of vital signs and adverse events. Participants were positioned to promote safety (e.g. positioning the participant in a corner or by a bed during balance exercise to prevent falling to the floor). Participants were educated on vital sign monitoring, dosing exercises to the appropriate intensity, and other safety considerations for completing home exercises. During weeks 7–12, participants received weekly check-in calls from a study team research assistant. |
| Batalik et al (2020) Czech Republic [43] | To focus on the use of the wrist heart rate monitor as a telerehabilitation (TR) device, define detected limitations, and compare the effect between home-based TR and regular outpatient CR methods related to physical fitness, quality of life, and training adherence. | IG: 5 (20% female) [56.5 years] CG: 4 (15.4% female) [57.7 years] | IG: Cardiac rehab CG: Regular outpatient training | Full telerehab Asynchronous | **Who provided:** Physiotherapist  **Where:** Patients in their own homes. Therapist in unknown location | 3 sessions/  week [80 min] x 12 weeks | **Tailoring:** Not specified **Modifications:** Not specified **Adherence:** 88.2% | - Web application - Wrist HR monitor - Mobile phone - Internet access - Educational booklet | Weekly telephone calls and feedback in the form of recommendations, advice, and motivation. |
| Wilson et al (2021) Australia [35] | To compare use of the Elements by Dynamic Neural Arts (EDNA) system with an active control (Graded Repetitive Arm Supplementary Program—GRASP training) group for stroke patients. | IG: 3 (30% female) [69.9 years] CG: 2 (28.6% female) [77.3 years] | IG: Stroke rehab CG: GRASP training | Full telerehab Asynchronous | **Who provided:** Occupational therapist  **Where:** Patients in their own homes. Therapist in unknown location | 3.5 sessions/  week [30 min] x 8 weeks | **Tailoring:** Not specified **Modifications:** Not specified **Adherence:** 80% | - Virtual reality (VR) - EDNA software - Touchscreen tablet | Weekly telephone calls to address questions/concerns. |
| van der Kolk et al (2019) Netherlands [55] | To evaluate the effectiveness of aerobic exercise—gamified and delivered at home, to promote adherence—on relieving motor symptoms in patients with Parkinson’s disease with mild disease severity who were on common treatment regimes. | IG: 23 (35.4% female) [59.3 years] CG: 27 (41.5% female) [59.4 years] | IG: Neuro rehab CG: Stretching, flexibility, and relaxation exercises | Hybrid telerehab Asynchronous | **Who provided:** Coach (physical therapist or research assistant)  **Where:** Patients in their own homes. Therapist in unknown location | ≥3 sessions/  week [45 min] x 26 weeks | **Tailoring:** Programs were personalized to the patient’s abilities to ensure all eligible patients could complete the program. Intensity was within a predetermined HR zone on the basis of their HR reserve **Modifications:** Patients were instructed to cycle at a target HR zone, which was gradually increased for goal setting as patients became fitter **Adherence:** 73% | - Customized tablet-based motivational app - Telephone for coaching - Stationary home-trainer cycle/bike enhanced with VR software and real-life videos - Chest-bound HR monitor | Cycling was chosen since it has a low risk of falling when patients exercise at home without physical supervision. Patients were instructed to report adverse events directly to their coach. |
| Song et al (2020) China [23] | To investigate the effects of telemonitored exercise rehabilitation on patients with coronary heart disease in China. | IG: 5 (10.4% female) [54.2 years] CG: 8 (16.7% female) [54.8 years] | IG: Cardiac rehab CG: Routine follow-up | Full telerehab Synchronous | **Who provided:** Researchers, which may include cardiologists or research assistants  **Where:** Patients in their own homes. Therapist in unknown location | 3-5 sessions/  week [30 min] x 26 weeks | **Tailoring:** Given exercise prescription according to CPET results **Modifications:** Not specified **Adherence:** Not specified | - Telemonitoring software - HR belt | Medical staff monitored patients’ exercise frequency, HR, and BP before and after exercise, investigated degree of fatigue after exercise, and communicated with patients weekly through text messaging and phone calls. |
| Snoek et al (2020) Europe: Netherlands, Denmark, Spain, Switzerland, France [53] | To assess whether a 6-month guided mobile cardiac rehabilitation program is an effective therapy for elderly patients who decline participation in cardiac rehabilitation. | IG: 20 (22.5% female) [72.4 years] CG: 14 (15.6% female) [73.6 years] | IG: Cardiac rehab CG: Usual care | Full telerehab Asynchronous | **Who provided:** Multidisciplinary team comprised of cardiologists, nurses, dieticians, and physiotherapists  **Where:** Patients in their own homes. Therapist in unknown location | 5 sessions/  week [≥30 min] x 26 weeks | **Tailoring:** The outcomes of the maximal exercise tests conducted during the first visit allowed the researchers to customize HR zones for each patient **Modifications:** Not specified **Adherence:** Not specified | - HR belt - Smartphone with a special mobile application | Participants were contacted by researchers weekly during month 1, every other week during month 2, and then monthly until 6 months. They could self-report adverse events during these check-ins. |
| Saitoh et al (2022) Japan [46] | To investigate the feasibility on remote CR support program in older patients with cardiac disease. | IG: 2 (33.3% female) [74 years] CG: 1 (20% female) [68 years] | IG: Cardiac rehab CG: Centre-based cardiac rehab | Full telerehab Asynchronous | **Who provided:** Physiotherapist  **Where:** Patients in their own homes. Therapist in unknown location | ≥3 sessions/  week [20-30 min] x 4 weeks | **Tailoring:** Not specified **Modifications:** Not specified **Adherence:** 30% | - Tablet computer - Remote real-time telemedicine system - Exercise videos - Pedometer - Logbook - Electrocardiogram (ECG) - Pulse oximeter - Electronic sphygmomanometer | ECG, pulse oximetry, and BP were recorded during telerehab sessions. It is presumed that the physiotherapist meeting weekly with the participants reviewed these. |
| Piraux et al (2019) Belgium [58] | To investigate feasibility and preliminary effects of telerehabilitation for people living with HIV and taking antiretroviral therapy. | IG: 3 (33.3% female) [47.2 years] CG: 3 (37.5% female) [46.1 years] | IG: Resistance training CG: Usual routine | Full telerehab Asynchronous | **Who provided:** Physiotherapist  **Where:** Patients in public fitness centers. Therapist in unknown location | 3 sessions/  week [50 min] x 6 weeks | **Tailoring:** Intensity was based on HR, 6-minute walk test (6MWT), and 1 repetition maximum **Modifications:** The exercise program was adapted by physiotherapists if necessary **Adherence:** 69% | - Website with all information needed to perform the training - Video with exercise instructions | A physiotherapist contacted participants weekly by telephone to check for adverse events. Patients could also contact this physiotherapist via the website or by phone at any time. |
| Piotrowicz et al (2019) Poland [56] | To assess whether potential improvements in quality of life outcomes after a 9-week home-based CR intervention in patients with heart failure translate into improvement in clinical outcomes during extended 12 to 24 months of follow-up, compared with usual care. | IG: 48 (11.3% female) [62.6 years] CG: 49 (11.5% female) [62.2 years] | IG: Cardiac rehab CG: Usual care | Hybrid telerehab Asynchronous | **Who provided:** Medical team comprised of physicians, physiotherapists, nurses, and a psychologist  **Where:** Patients in their own homes. Therapist in unknown location | 5 sessions/  week x 8 weeks | **Tailoring:** The medical team tailored each patient’s rehabilitation program **Modifications:** Not specified **Adherence:** 78% | - Special remote device for supervised exercise training monitored with tele-ECG - Data transmission set via a mobile telephone - Monitoring center capable of receiving and storing patients’ medical data | Tele-ECG for telerehab participants during sessions. The patients who had cardiovascular implantable electronic devices also had information from those devices transmitted remotely. |
| Peng et al (2018) China [24] | To examine the effect of our telehealth exercise training program on health outcomes in patients with heart failure in China. | IG: 21 (42.9% female) [not reported] CG: 19 (38.8% female) [not reported] | IG: Cardiac rehab CG: Usual care | Full telerehab Synchronous | **Who provided:** Multidisciplinary team comprised of physiotherapists, cardiac nurses, and psychiatric nurses  **Where:** Patients in their own homes. Therapist in unknown location | 3-5 sessions/  week [20-30 min] x 8 weeks | **Tailoring:** Based on HR. The target training HR was 40-70% of the HR reserve plus the resting HR **Modifications:** Physiotherapists adjusted the training intensity according to each individual’s condition **Adherence:** Not specified | - Printed brochure - Online webcam communication - Supervision using QQ and WeChat - Instant messaging | An education session on exercise training was held prior to discharge. The exercise intensity was low to moderate and based on patients’ personal conditions. Patients’ caregivers were asked to accompany them during the training sessions in case of an emergency. Each patient was given a HR monitor and was instructed to wear it during exercise and to report his or her real-time HR so that the rehabilitation doctors could adjust the training intensity accordingly. The patients were asked to report to the doctor and stop the exercises immediately if they experienced signs or symptoms of physical distress. |
| Paul et al (2019) UK [57] | To examine the feasibility of a trial to evaluate web-based physiotherapy compared to a standard home exercise program in people with multiple sclerosis. | IG: 45 (71.1% female) [55.6 years] CG: 45 (82.2% female) [56.5 years] | IG: Neuro rehab CG: Printed exercise sheets | Full telerehab Asynchronous | **Who provided:** Physiotherapist  **Where:** Patients in their own homes. Therapist in unknown location. | 2 sessions/  week x 26 weeks | **Tailoring:** The intensity of exercises was individualized to meet the participants’ needs **Modifications:** The physiotherapist reviewed electronic exercise diaries every two weeks and remotely altered programs in response to participants’ comments **Adherence:** 40-63% | - Website containing exercise videos and educational materials (www.webbasedphysio.com) | Self-report questionnaire included a space to report healthcare resource use, physiotherapist time, general practitioner visits, nurse visits, other multiple sclerosis or outpatient review, accident and emergency attendance and hospital stay. |
| Ozturk et al (2022) Turkey [44] | To examine the effects of exercise training through telerehabilitation applied during COVID-19 isolation period on overweight and obese individuals on physical fitness and quality of life. | IG: 21 (57.1% female) [41.1 years] CG: 20 (42.9% female) [40.9 years] | IG: Body stabilization-based mat exercises and breathing exercises CG: Usual routine and 1 remote meeting on exercise and breathing exercises | Full telerehab Synchronous | **Who provided:** Physiotherapist  **Where:** Patients in their own homes. Therapist in unknown location | 3 sessions/  week [45 min] x 6 weeks | **Tailoring:** Not specified **Modifications:** Degree of difficulty was increased approximately every 2 weeks. Exercises were modified for participants who had difficulty **Adherence:** 100% | - Computer program - Exercise mat | Participants were given details for who to contact during emergencies. |
| Øra et al (2020) Norway [38] | To pilot a definitive randomized controlled trial of speech-language telerehabilitation in poststroke aphasia in addition to usual care with regard to recruitment, drop-outs, and language effects. | IG: 32 (40.6% female) [64.7 years] CG: 30 (26.7% female) [65 years] | IG: Stroke rehab CG: Usual care | Full telerehab Synchronous | **Who provided:** Speech-language pathologist  **Where:** Patients in their own homes or admitted to secondary rehabilitation centers. Therapist in unknown location | 5 sessions/  week [60-120 min] x 4 weeks | **Tailoring:** The therapy was tailored to the individual participant’s language impairment, needs, and goals in all language modalities (reading, writing, spoken language, and auditory comprehension) **Modifications:** Not specified **Adherence:** Not specified | - Newcastle University Aphasia Therapy Resources - Computer training program ("Lexia") - Laptop - External speaker - Wide-angle web camera - Videoconferencing software ("Cisco") - Texts, maps, and pictures | Encrypted software was used to mitigate data privacy events. Participants were given a wide-angle web camera to enable a full view during videoconferencing sessions. |
| Hong et al (2023) China [25] | To evaluate the effectiveness of a six-week home-based program on the pain and function, as well as isokinetic muscle strength of the knee joint in patients with patellofemoral pain. | IG: 19 (47.4% female) [31.8 years] CG: 19 (42.1% female) [32.3 years] | IG: Strength-based exercises CG: Health education on patellofemoral pain | Full telerehab  Asynchronous | **Who provided:** Physiotherapist  **Where:** Patients in their own homes. Therapist communicated via WeChat | 3 sessions/  week x 6 weeks | **Tailoring:** The program was adjusted every two weeks through one-to-one guidance by physiotherapists **Modifications:** Physiotherapists adjusted the load of exercises if necessary **Adherence:** 93% | - Videos with exercise instructions - Exercise booklets | Participants were allowed to use temporary pain medications as necessary. |
| Liu et al (2022) China [26] | To develop an immersive VR-based puzzle game and explore its effectiveness, feasibility, and safety in elderly stroke patients with cognitive dysfunction. | IG: 15 (60% female) [74.9 years] CG: 15 (52.3% female) [73.4 years] | IG: Stroke rehab CG: Traditional cognitive training | Full telerehab Synchronous | **Who provided:** Therapist **Where:** Patients in their own homes. Therapists in a room at the Department of Rehabilitation Assessment, Shanghai Fourth Rehabilitation Hospital | 6 sessions/  week x 6 weeks | **Tailoring:** Investigators selected games according to patients’ interests **Modifications:** Difficulty was gradually adjusted from simple to complex **Adherence:** Not specified | - Head-mounted display and sensor | Treatment was immediately stopped if participants experienced any intolerable discomfort. |
| Pastana Ramos et al (2023) Brazil [41] | To assess the feasibility and efficacy of a telerehabilitation program for people with Parkinson’s disease living in an underrepresented community of the Brazilian Amaz. | IG: 8 (50% female) [60.7 years] CG: 11 (45.6% female) [58.6 years] | IG: Neuro rehab  CG: Booklet-based exercise program | Full telerehab Synchronous | **Who provided:** Physiotherapist **Where:** Patients in their own homes. Therapist in unknown location | 3 sessions/  week [60 min] x 4 weeks | **Tailoring:** Not specified **Modifications:** The intensity level was reduced if patients reported fatigue **Adherence:** Not specified | - Teleconference web platforms (i.e., "WhatsApp", "Google Meet") - Smartphone - Tablet - Computer | Only patients with good performance in a baseline 6MWT could participate in the intervention. In-person caregiver supervision during telerehab sessions was required. All sessions included a warm-up and cool-down. Only moderately intense exercises were performed. The physiotherapist checked feedback and adverse events once a week via telephone call. |
| Master et al (2023) USA [30] | To examine the feasibility and acceptability of a wearable device and telehealth counseling physical activity intervention early after lumbar spine surgery. | IG: 8 (50% female) [65.4 years] CG: 8 (50% female) [63 years] | IG: Aerobic exercise CG: Usual care | Full telerehab Asynchronous | **Who provided:** Physiotherapist **Where:** Patients in their own homes. Therapist in unknown location | 1 session/  week x 8 weeks | **Tailoring:** Not specified  **Modifications:** Participants progressed when they met their walking goal on 4 out of 7 days. If they did not, the therapists would discuss barriers and adjust the program accordingly **Adherence:** 88% | - Wearable device (i.e., "Fitbit") - Daily step goal tracking sheet  - Videoconferencing software (i.e., "Zoom") | Adverse events were assessed by study personnel at follow up time points. |
| Nuevo et al (2023) Spain [51] | To demonstrate the safety and efficacy of an interactive telerehabilitation system (ReHub®) to guide and provide feedback during exercise in the postoperative period of a fast-track total knee arthroplasty program. | IG: 23 (39.2% female) [68.3 years] CG: 22 (63.6% female) [68.8 years] | IG: Lower limb muscle contraction exercises CG: Booklet-based exercise program | Full telerehab Synchronous | **Who provided:** Physiotherapist **Where:** Patients in their own homes. Therapist in unknown location | 7 sessions/  week x 4 weeks | **Tailoring:** Physiotherapists tailored the rehab program to each patient’s condition **Modifications:** The ReHub® platform algorithms delivered real-time biofeedback through an animated talking coach to suggest corrections **Adherence:** 86% | - Leaflet with exercises - Rehub® platform - Accelerometer - Gyroscope - Magnetometer - Strain gauge - Resistance bands | The physiotherapists provided an at-home training session for the Rehub® platform and followed up in-person two weeks later. |
| Lundgren et al (2023) Norway [39] | To evaluate the feasibility of a 3-month real-time, home-based telerehabilitation, high-intensity exercise program for congestive heart failure patients who are either unable or unwilling to participate in standard outpatient cardiac rehabilitation and to explore outcomes of self-efficacy and physical fitness at 3 months post-intervention. | IG: 31 (25.8% female) [67.6 years] CG: 30 (10% female) [67.7 years] | IG: Cardiac rehab CG: Usual care | Full telerehab  Synchronous | **Who provided:** Physiotherapist specialized in cardiac rehab **Where:** Patients in their own homes. Therapist in unknown location | 2 sessions/  week x 12 weeks | **Tailoring:** The program was adapted if participation was prevented by illness or injury **Modifications:** Not specified **Adherence:** 38.7% of participants were adherent (attended ≥80% of sessions), 41.9% were partially adherent (attended 21-79% of sessions), and 19.4% were non-adherent (attended ≤20% of sessions) | - Apple iPad Air 2 - 4G mobile network SIM card (for those without wireless internet facilities at home) | Addresses and telephone numbers for all participants were made available, enabling the physical therapist to immediately call for professional medical help in the event of an emergency. |
| Guo et al (2023) China [27] | To design a remote intelligent rehabilitation training system based on wearable devices and human-computer interaction training tasks, and to evaluate the effectiveness and safety of the remote rehabilitation training system for nonphysician- supervised motor rehabilitation training of patients with stroke through a clinical trial study. | IG: 60 (28.3% female) [56.3 years] CG: 60 (28.3% female) [55.8 years] | IG: Stroke rehab CG: Routine occupational and physical therapy training | Full telerehab Asynchronous | **Who provided:** Rehabilitation physician **Where:** Patients and rehabilitation physician could be located in the training hall, community, or home | 14 sessions/  week x 3 weeks | **Tailoring:** The software modified the games and difficulty level according to the patient’s recovery status **Modifications:** The software tracks strength, speed, distance, and other movement features during rehabilitation, providing visual or auditory feedback to help patients adjust. Results are uploaded to a server, allowing physicians to modify the exercise plan based on rehabilitation scores. The updated prescription is automatically integrated into the patient’s program, with daily adjustments **Adherence:** Not specified | - Remote rehabilitation system that integrated training equipment hardware, communication training games, training software, remote rehabilitation management platform, and a mobile application - Training gloves that contain 1 inertial measurement unit and 5 flex sensors - 9-axis (3-axis accelerometer, 3-axis angular velocity meter, 3-axis magnetometer) motion sensors | During the training process, the rehabilitation physician or therapist was next to the patient to ensure the patient’s safety. |
| Park et al (2023) South Korea [48] | To investigate the effect of hospital-home linked rehabilitation therapy using an augmented reality-based digital health care system (UINCARE Home+) in postoperative patients with breast cancer. | IG: 50 (not reported) [42.6 years] CG: 50 (not reported) [47.3 years] | IG: Cancer rehab CG: Brochure-based rehab | Full telerehab Asynchronous | **Who provided:** Physician **Where:** Patients in their own homes. Physicians in unknown location | 7 sessions/  week x 8 weeks | **Tailoring:** The exercise level was determined according to the results obtained over the first 4 weeks **Modifications:** Participants received instant feedback (sound and circle on the screen). After the exercise, task performance parameters (repetitions and movement accuracy) were recorded in real time on the internet server and the physicians provided feedback **Adherence:** 64.4% | - UINCARE Home+ rehabilitation system - Xbox One Kinect | There were regularly scheduled follow-ups where outcomes such as pain were assessed. |
| Goffredo et al (2023) Italy [49] | To expand the scope of our previously published research on the impact of telerehabilitation on quality of life in a multiple sclerosis sample, testing the impact of this type of intervention in a larger sample of neurological patients also including Parkinson's disease individuals on postural balance. | IG: 65 (55.4% female) [58.1 years] CG: 67 (55.2% female) [61.1 years] | IG: Neuro rehab CG: Conventional rehab | Full telerehab Asynchronous | **Who provided:** Therapist **Where:** Patients in their own homes. Therapist in unknown location | 5 sessions/  week x 6-8 weeks | **Tailoring:** The therapists customized the rehabilitation protocol according to the characteristics and needs of the patient **Modifications:** Not specified **Adherence:** Not specified | - Virtual Reality Rehabilitation System Tablet home telerehabilitation system - Inertial sensors | Not specified |
| Polo et al (2023) USA [31] | To adapt a standard pulmonary rehabilitation program to be delivered via telehealth. | IG: 111 (60.3% female) [66.9 years] CG: 98 (58.2% female) [66 years] | IG: Pulmonary rehab CG: Standard pulmonary rehab | Full telerehab Synchronous | **Who provided:** Respiratory therapist **Where:** Patients in their own homes or community center. Therapist in unknown location | 2 sessions/  week [60 min] x 8 weeks | **Tailoring:** The respiratory therapist developed an individualized exercise program for each participant based on exercise capacity **Modifications:** Not specified **Adherence:** 59.2% | - Full-size recumbent bicycle - Weights - Stretch bands - Vital sign monitor - Tablet computer with Wi-Fi card - Nonin watch - Exercise peddler - Videoconferencing software (i.e., "Zoom") | Before the first session, participants needed to be medically cleared to exercise by a pulmonologist. A study-specific Community Advisory Board (included patients, caregivers, directors of pulmonary rehab clinics, and clinicians) was created and provided recommendations on study protocols, safety features, and equipment functionality. Participants received a Nonin watch that transmitted vital signs directly to the platform for continuous monitoring during the sessions. A pulmonologist was on-call during each session in case of an emergency. |
| Swarnakar et al (2023) India [47] | To delve deeper into the efficacy of telerehabilitation in addressing the rehabilitation needs of individuals with spinal cord injury. | IG: 15 (20% female) [28.2 years] CG: 15 (6.7% female) [26.3 years] | IG: Neuro rehab CG: Usual care | Full telerehab Synchronous | **Who provided:** Physiotherapists and occupational therapists **Where:** Patients in their own homes. Therapist in unknown location | 2 sessions/  week [60 min] x 8 weeks | **Tailoring:** Physiotherapy techniques were modified according to the patient’s needs **Modifications:** Not specified **Adherence:** 100% | Not specified | A data and safety monitoring board was formed and could decide to withdraw or remove a study participant if there were any serious adverse events or safety concerns. The board members were accessible by telephone to all participants throughout the study. |
| Pak et al (2023) USA [32] | To compare clinical outcomes between digital physical therapy and conventional in-person physical therapy in patients with chronic shoulder pain. | IG: 24 (58.5% female) [49.7 years] CG: 19 (46.3% female) [50.8 years] | IG: Mobility, stretching, and strengthening exercises CG: Conventional in-person physical therapy | Full telerehab Asynchronous | **Who provided:** Licensed physical therapist with clinical doctorate credentials **Where:** Patients in their own homes. Therapist in unknown location | 3 sessions/  week [20 min] x 8 weeks | **Tailoring:** An initial onboarding video call consisting of a clinical evaluation led to a program tailored to the participant’s needs **Modifications:** The physical therapist could access motion data asynchronously and adjust the program accordingly **Adherence:** Not specified | - FDA-listed class II medical device consisting of 3 inertial motion trackers - Tablet with a mobile app - Cloud-based portal | Patient education via smartphone app which included articles on anatomy, physiology, pain reconceptualization, active coping skills, exercise, and fear-avoidance behaviors. |
| Cerdan de las Heras et al (2022) Denmark [50] | To motivate patients with sarcoidosis to increase their physical activity with the use of telerehabilitation and analyze the effect and usefulness of telerehabilitation on exercise capacity and quality of life in patients with sarcoidosis. | IG: 5 (33.3% female) [56.1 years] CG: 6 (40% female) [51.6 years] | IG: Aerobic and strengthening exercises CG: Usual care | Full telerehab Asynchronous | **Who provided:** Virtual Autonomous Physiotherapist Agent (VAPA) **Where:** Patients in their own homes. Therapist in unknown location | 12 weeks | **Tailoring:** The physiotherapist individualized the telerehabilitation program based on baseline 6MWT and an interview focused on participant daily activity **Modifications:** Online meetings were scheduled with the participant to adjust the training program **Adherence:** 64% | - VAPA (service platform for therapists to create customized rehabilitation programs, video consultations, e-learning packages, physical exercise programs, online questionnaires, patient digital files, and chat directly with patients) - Kit composed of a smart tablet and biometric sensor | Not specified |
| Plaza et al (2023) Australia [36] | To determine if a six week exercise program for adults with burn injuries, delivered via home-based telerehabiliation, was as effective as (or non-inferior to) in-person programs with respect to patient related clinical outcomes. | IG: 7 (30.4% female) [48.4 years] CG: 7 (31.8% female) [45.1 years] | IG: Burn rehab CG: In-person exercise program | Full telerehab Synchronous | **Who provided:** Physical therapist with burn experience **Where:** Patients in their own homes. Therapist in unknown location | 0.5-2 sessions/  week [30-60 min] x 6 weeks | **Tailoring:** Tailored to the patient’s body area underlying the burn injury **Modifications:** Not specified **Adherence:** 44% | - Burn injury-specific education - Home exercise program - Exercise sheets - Burn exercise DVD | The physiotherapist had burn experience and provided patients with educational materials specific to their burn injury. |
| Alhusayni et al (2023) Scotland [52] | To evaluate the acceptability and feasibility of an individualized program of upper-limb rehabilitation that is delivered via an online rehabilitation platform for inpatient stroke survivors. | IG: 7 (53.8% female) [66.8 years] CG: 8 (61.5% female) [69 years] | IG: Stroke rehab CG: Usual care | Full telerehab Asynchronous | **Who provided:** Physiotherapists or assistant physiotherapists  **Where:** Patients in their own homes. Therapist in unknown location | 5 sessions/week [30 min] x 4 weeks | **Tailoring:** The individualized program was based on clinical assessment, the participant’s goals, and their level of upper-limb function. The exercise dose (duration, frequency, and intensity) was based on the participant’s level of functional ability **Modifications:** Physiotherapists reviewed participants’ progress once a week and made any necessary changes to their program. Participants were able to contact the research team during the study to ask any questions or to request a change in their program **Adherence:** 38% | - Leaflet from Chest Heart & Stroke Scotland about exercise and physical activity - Tablet and/or internet (if participants did not have an internet-enabled device) | Participants were able to contact the research team during the study to ask any questions or to request a change in their program. In addition, an aphasia-friendly version of the advice section on the website was available. |
