# Supplementary material for: The Safety of Telerehabilitation: Systematic Review
Source: JMIR Rehabil Assist Technol. 2025 Jul 9;12:e68681. doi: 10.2196/68681 (PMC12266302; doi:10.2196/68681)
Supplement: Multimedia Appendix 3 [file rehab-v12-e68681-s003.docx]

| **Study/Year/Country** | **Adverse Events - Intervention Group** | | | | | | **Adverse Events - Control Group** | | | | | |
| --- | --- | --- | --- | --- | --- | --- | --- | --- | --- | --- | --- | --- |
|  | **Events/**  **Participants** | **Events/**  **Sessions** | **Severity** | **Type** | **Relatedness** | **Description** | **Events/**  **Participants** | **Events/**  **Sessions** | **Severity** | **Type** | **Relatedness** | **Description** |
| Keteyian et al (2021) USA [28] | 1/26 | 1/468 | Non-serious | Physical | Related | Fall (1) | 0/21 | 0/378 | Not specified | Not specified | Not specified | Not specified |
| Jarbandhan et al (2022) Suriname [45] | 0/20 | 0/240 | Not specified | Not specified | Not specified | Not specified | 0/10 | 0/100 | Not specified | Not specified | Not specified | Not specified |
| Hwang et al (2017) Australia [65] | 6/24 | 6/480 | Minor | Physical | Not specified | Angina (3), diaphoresis (1), palpitations (2) | 2/29 | 2/96 | Minor | Not specified | Not specified | Diaphoresis (2) |
| Hume et al (2022) UK [37] | 0/7 | 0/568 | Not specified | Not specified | Not specified | Not specified | 0/5 | Not applicable | Not specified | Not specified | Not specified | Not specified |
| Gehring et al (2018) Netherlands [54] | 0/23 | 0/1656 | Not specified | Not specified | Not specified | Not specified | 0/11 | Not applicable | Not specified | Not specified | Not specified | Not specified |
| Fioratti et al (2022) Brazil [40] | 0/31 | 0/744 | Not specified | Not specified | Not specified | Not specified | 0/33 | Not applicable | Not specified | Not specified | Not specified | Not specified |
| Donkers et al (2020) Canada [42] | 0/32 | 0/1664 | Not specified | Not specified | Unrelated | Not specified | 0/16 | 0/832 | Not specified | Not specified | Not specified | Not specified |
| Cox et al (2022) Australia [34] | 0/68 | 0/1088 | Not specified | Not specified | Not specified | Not specified | 4/67 | 4/1072 | Not specified | Not specified | Unrelated (1), possibly related (1), not specified (2) | Respiratory hospitalization during rehabilitation period (2), blurred vision requiring hospitalization and testing (1), hypotensive (1) |
| Chen et al (2021)  China [22] | 0/40 | 0/Not specified | Not specified | Not specified | Not specified | Not specified | 0/40 | Not applicable | Not specified | Not specified | Not specified | Not specified |
| Capin et al (2022) USA [29] | 0/28 | 0/336 | Not specified | Not specified | Not specified | Not specified | 17/13 | Not applicable | Severe (1), moderate (4), minor (12) | Not specified | Not specified | Hospitalization (1), not specified (16) |
| Batalik et al (2020) Czech Republic [43] | 0/25 | 0/900 | Not specified | Not specified | Not specified | Not specified | 1/26 | 1/936 | Not specified | Hospitalization/cardiac | Not specified | Admission to hospital for cardiac symptoms (1) |
| Wilson et al (2021) Australia [35] | 0/10 | 0/280 | Not specified | Not specified | Not specified | Not specified | 0/7 | 0/196 | Not specified | Not specified | Not specified | Not specified |
| van der Kolk et al (2019) Netherlands [55] | 27/65 | 27/3900 | Non-serious (24), serious (3) | Not specified | Unrelated (20), potentially related (7) | Arthralgia/back pain (2), palpitations (4), vasovagal reaction during the baseline visit (2), vestibilar disorder (1), vasovagal collapse (1), knee injury during gardening that required surgery (1), acute positional vertigo which was eventually diagnosed as Benign Paroxysmal Positional Vertigo (1), other (15) | 29/65 | 29/5070 | Non-serious (25), serious (4) | Physical | Unrelated (25), potentially related (4) | Supraventricular tachycardia (1), atrial fibrillation (1), vasovagal reaction during the baseline visit (2), transient ischemic attack (2), neuralgia (1), hip fracture (1), fall related injury (2), severe dyskinesias after suprathreshold dose levodopa in a patient with deep brain stimulation (1), arthralgia/back pain (4), fall related injury (2), arrhythmias (2), cardiac disorders (1), injury, poisoning and procedural complications (3), other (6) |
| Song et al (2020) China [23] | 0/48 | 0/5875 | Not specified | Not specified | Not specified | Not specified | 0/48 | 0/4992 | Not specified | Not specified | Not specified | Not specified |
| Snoek et al (2020) Europe: Netherlands, Denmark, Spain, Switzerland, France [53] | 12/89 | 12/10680 | Serious | Not specified | Not specified | Near sudden cardiac death (1), acute coronary syndrome (4), chronic coronary syndrome (6), endocarditis (1) | 10/90 | Not applicable | Not specified | Not specified | Not specified | Near sudden cardiac death (1), acute coronary syndrome (3), chronic coronary syndrome (2), pacemaker (2), percutaneous coronary intervention (1), dyspnea (1) |
| Saitoh et al (2022) Japan [46] | 0/6 | 0/72 | Not specified | Not specified | Not specified | Not specified | 0/5 | 0/20 | Not specified | Not specified | Not specified | Not specified |
| Piraux et al (2019) Belgium [58] | 0/9 | 0/162 | Not specified | Not specified | Not specified | Not specified | 0/8 | Not applicable | Not specified | Not specified | Not specified | Not specified |
| Piotrowicz et al (2019) Poland [56] | 63/425 | 63/17000 | Non-serious | Physical | Unrelated (51), not specified (12) | Dyspnea/tachypnea >20 breaths/min (9), syncope (3), weight gain of at least 1.8 kg during 1-3 days (8), complex ventricular arrhythmia (7), resting heart rate >100 beats per minute (3), angina (2), decrease in New York Heart Association class (3), supraventricular tyachycardia, atrial flutter/atrial fibrillation (8), signs and symptoms of heart failure (2), necessity for urgent hospitalization (7), other (11) | 2/425 | Not applicable | Severe | Unknown (1), cardiac (1) | Not specified | Death of unknown cause (1), suddent cardiac death (1) |
| Peng et al (2018) China [24] | 0/49 | 0/1568 | Not specified | Not specified | Not specified | Not specified | 0/49 | Not applicable | Not specified | Not specified | Not specified | Not specified |
| Paul et al (2019) UK [57] | 27/45 | 27/2340 | Not specified | Physical | Unrelated | Falls, skin reactions | 33/45 | 33/2340 | Not specified | Physical | Unrelated | Falls, skin reactions |
| Ozturk et al (2022) Turkey [44] | 0/21 | 0/378 | Not specified | Not specified | Not specified | Not specified | 0/20 | Not applicable | Not specified | Not specified | Not specified | Not specified |
| Øra et al (2020) Norway [38] | 0/32 | 0/512 | Not specified | Not specified | Not specified | Not specified | 0/30 | Not applicable | Not specified | Not specified | Not specified | Not specified |
| Hong et al (2023) China [25] | 0/19 | 0/342 | Not specified | Not specified | Not specified | Not specified | 0/19 | Not applicable | Not specified | Not specified | Not specified | Not specified |
| Liu et al (2022) China [26] | 7/15 | 7/540 | Mild | Sensory and physical | Not specified | Dizziness (2), dry eyes (2), eye fatigue (3) | 0/15 | 0/540 | Not specified | Not specified | Not specified | Not specified |
| Pastana Ramos et al (2023) Brazil [41] | 6/8 | 6/96 | Mild | Physical and infection-related | Unrelated | Flu symptoms (2), pain (3), tiredness (1) | 5/11 | 5/132 | Mild | Physical and infection-related | Not specified | Flu symptoms (3), pain (1), COVID-19 infection (1) |
| Master et al (2023) USA [30] | 0/8 | 0/64 | Not specified | Not specified | Not specified | Not specified | 0/8 | Not applicable | Not specified | Not specified | Not specified | Not specified |
| Nuevo et al (2023) Spain [51] | 3/23 | 3/644 | Moderate | Physical | Unrelated (2), related (1) | Functional overload with pain and swelling (1), not specified (2) | 3/22 | 3/616 | Not specified | Not specified | Not specified | Not specified |
| Lundgren et al (2023) Norway [39] | 22/31 | 22/744 | Not specified | Not specified | Unrelated | Hypoglycemia (1), not specified (21) | 13/30 | Not applicable | Not specified | Not specified | Not specified | Not specified |
| Guo et al (2023) China [27] | 22/60 | 22/3780 | Not specified | Not specified | Unrelated | Not specified | 28/60 | 28/5040 | Not specified | Not specified | Not specified | Not specified |
| Park et al (2023) South Korea [48] | 0/50 | 0/2800 | Not specified | Not specified | Not specified | Not specified | 0/50 | 0/2800 | Not specified | Not specified | Not specified | Not specified |
| Goffredo et al (2023) Italy [49] | 0/65 | 0/2275 | Not specified | Not specified | Not specified | Not specified | 0/67 | 0/2345 | Not specified | Not specified | Not specified | Not specified |
| Polo et al (2023) USA [31] | 0/111 | 0/1776 | Not specified | Not specified | Unrelated | Not specified | 0/98 | 0/1568 | Not specified | Not specified | Not specified | Not specified |
| Swarnakar et al (2023) India [47] | 0/15 | 0/60 | Not specified | Not specified | Not specified | Not specified | 0/15 | Not applicable | Not specified | Not specified | Not specified | Not specified |
| Pak et al (2023) USA [32] | 0/41 | 0/984 | Not specified | Not specified | Unrelated | Not specified | 0/41 | 0/615 | Not specified | Not specified | Not specified | Not specified |
