# Supplementary material for: The Safety of Telerehabilitation: Systematic Review
Source: JMIR Rehabil Assist Technol. 2025 Jul 9;12:e68681. doi: 10.2196/68681 (PMC12266302; doi:10.2196/68681)
Supplement: Multimedia Appendix 4 [file rehab-v12-e68681-s004.pdf]

Risk of bias domains

Study	D1	D2	D3	D4	D5	Overall
Keteyian et al (2021) USA [28]	-	+	+	X	+	X
Jarbandhan et al (2022) Suriname [45]	+	-	+	-	+	-
Hwang et al (2017) Australia [65]	+	+	+	-	+	-
Hume et al (2022) UK [37]	+	+	+	+	+	+
Gehring et al (2018) Netherlands [54]	+	+	+	+	+	+
Fioratti et al (2022) Brazil [40]	+	+	+	+	+	+
Donkers et al (2020) Canada [42]	+	+	+	+	+	+
Cox et al (2022) Australia [34]	+	+	+	+	+	+
Chen et al (2021) China [22]	+	+	+	+	+	+
Capin et al (2022) USA [29]	+	+	+	+	+	+
Batalik et al (2020) Czech Republic [43]	+	+	+	+	+	+
Wilson et al (2021) Australia [35]	+	+	+	+	+	+
van der Kolk et al (2019) Netherlands [55]	+	+	+	+	+	+
Song et al (2020) China [23]	+	+	+	+	+	+
Snoek et al (2020) Europe: Netherlands, Denmark, Spain, Switzerland, France [53]	+	+	+	+	-	-
Saitoh et al (2022) Japan [46]	+	+	+	-	+	-
Piraux et al (2019) Belgium [58]	+	+	+	-	+	-
Piotrowicz et al (2019) Poland [56]	+	+	-	+	+	-
Peng et al (2018) China [24]	+	+	+	+	+	+
Paul et al (2019) UK [57]	+	+	+	+	+	+
Ozturk et al (2022) Turkey [44]	+	+	+	-	+	-
Øra et al (2020) Norway [38]	+	+	+	+	+	+
Hong et al (2023) China [25]	+	+	+	+	+	+
Liu et al (2022) China [26]	+	+	+	+	+	+
Pastana Ramos et al (2023) Brazil [41]	+	+	+	+	+	+
Master et al (2023) USA [30]	+	+	+	+	+	+
Nuevo et al (2023) Spain [51]	+	+	+	+	+	+
Lundgren et al (2023) Norway [39]	+	+	+	-	+	-
Guo et al (2023) China [27]	+	+	+	+	+	+
Park et al (2023) South Korea [48]	+	+	+	+	+	+
Goffredo et al (2023) Italy [49]	+	+	+	+	+	+
Polo et al (2023) USA [31]	+	+	-	+	+	-
Swarnakar et al (2023) India [47]	+	+	+	+	+	+
Pak et al (2023) USA [32]	+	+	+	+	+	+
Cerdan de las Heras et al (2022) Denmark [50]	+	+	+	+	+	+
Plaza et al (2023) Australia [36]	+	+	+	+	+	+
Alhusayni et al (2023) Scotland [52]	+	+	+	X	+	X

Domains:
D1: Bias arising from the randomization process.
D2: Bias due to deviations from intended intervention.
D3: Bias due to missing outcome data.
D4: Bias in measurement of the outcome.
D5: Bias in selection of the reported result.

Judgement
X High
- Some concerns
+ Low
